# Functional hypoxia reduces mitochondrial calcium uptake^☆^

**DOI:** 10.1016/j.redox.2024.103037

**Published:** 2024-01-17

**Authors:** Chris Donnelly, Timea Komlódi, Cristiane Cecatto, Luiza H.D. Cardoso, Anne-Claire Compagnion, Alessandro Matera, Daniele Tavernari, Olivier Campiche, Rosa Chiara Paolicelli, Nadège Zanou, Bengt Kayser, Erich Gnaiger, Nicolas Place

**Affiliations:** a Institute of Sport Sciences, University of Lausanne, Lausanne, Switzerland; b Oroboros Instruments, Innsbruck, Austria; c Department of Biomedical Sciences, University of Lausanne, Lausanne, Switzerland; d Department of Computational Biology, University of Lausanne, Lausanne, Switzerland; e Swiss Institute of Bioinformatics, Lausanne, Switzerland; f Swiss Cancer Centre Léman, Lausanne, Switzerland

**Keywords:** Respirometry, Membrane potential, Skeletal muscle, Exercise, Coenzyme Q

## Abstract

Mitochondrial respiration extends beyond ATP generation, with the organelle participating in many cellular and physiological processes. Parallel changes in components of the mitochondrial electron transfer system with respiration render it an appropriate hub for coordinating cellular adaption to changes in oxygen levels. How changes in respiration under functional hypoxia (i.e., when intracellular O_2_ levels limit mitochondrial respiration) are relayed by the electron transfer system to impact mitochondrial adaption and remodeling after hypoxic exposure remains poorly defined. This is largely due to challenges integrating findings under controlled and defined O_2_ levels in studies connecting functions of isolated mitochondria to humans during physical exercise. Here we present experiments under conditions of hypoxia in isolated mitochondria, myotubes and exercising humans. Performing steady-state respirometry with isolated mitochondria we found that oxygen limitation of respiration reduced electron flow and oxidative phosphorylation, lowered the mitochondrial membrane potential difference, and decreased mitochondrial calcium influx. Similarly, in myotubes under functional hypoxia mitochondrial calcium uptake decreased in response to sarcoplasmic reticulum calcium release for contraction. In both myotubes and human skeletal muscle this blunted mitochondrial adaptive responses and remodeling upon contractions. Our results suggest that by regulating calcium uptake the mitochondrial electron transfer system is a hub for coordinating cellular adaption under functional hypoxia.

## 1 Introduction

Mitochondria use molecular oxygen (O_2_) as the terminal electron acceptor to generate the protonmotive force for phosphorylation of ADP [1]. O_2_ consumption is adjusted in response to changes in cellular ATP demand [2]. This is exemplified during intense muscle contractions which can increase O_2_ consumption up to 20-fold of that at rest [3]. Mitochondrial ATP regeneration by oxidative phosphorylation depends on the supply of O_2_ and ADP [4]. The state of ‘functional hypoxia’ describes when O_2_ levels limit mitochondrial respiration [5,6]. Although restricted to a very low intracellular *p*_O2_, functional hypoxia can occur during physiological activity [[7], [8], [9], [10]]), under conditions of ambient hypoxia [11], or during ischemia [12].

Although the O_2_ kinetics (i.e., dependence on O_2_ partial pressure) of oxidative phosphorylation have been resolved [13,14], there is considerable interest in understanding if and how O_2_ controls other components and functions of the electron transfer system [[15], [16], [17]]. This interest has been stimulated by findings showing functional hypoxia and impairments in mitochondrial respiration are hallmarks of many metabolic diseases [[18], [19], [20], [21], [22]] and that mitochondrial signals influence numerous cellular processes [[15], [16], [17],^23^]. Signals of particular interest are mitochondrial redox states [17,24] and the mitochondrial membrane potential difference (mt-membrane potential difference or Δ*Ψ*_mt_; [12]) which exert an influence on processes involved in cellular adaptions and physiological outcomes [15]. These processes include alterations in metabolite concentrations [25], mitochondrial reactive oxygen species production [26], the import of proteins into mitochondria [27], and the shaping of cellular calcium (Ca^2+^) signals [28].

Understanding the mechanisms through which functional hypoxia regulates biological outcomes has proved challenging, largely, due to the difficulty to integrate findings under well-controlled and defined O_2_ levels in models varying from isolated mitochondria to intact humans [5]. Nonetheless, the potential for components of the electron transfer system to play key roles in relaying signals for O_2_ sensing are highlighted by the rapid regulation of the system under functional hypoxia [24]. For example, at the onset of functional hypoxia (at only 5 % limitation of respiration by O_2_) there is a steep decline in cytochrome *c* oxidation state [24]. These parallel changes in components of the electron transfer system with respiration render it an appropriate hub for coordinating cellular adaption. How changes in respiration under hypoxia are relayed by the electron transfer system to elicit mitochondrial adaption after hypoxic exposure remains poorly defined. We hypothesized that changes in the mitochondrial electron transfer system could influence key processes that control cellular adaption under hypoxia.

Here we report experiments under conditions of functional hypoxia in carefully chosen and relevant models: isolated mitochondria, myotubes and muscle from exercising humans, and propose that O_2_ control of the mitochondrial electron transfer system reduces muscle mitochondrial Ca^2+^ uptake and thereby regulates mitochondrial adaptive remodeling upon physical exercise.

## 2 Results

### 2.1 Functional hypoxia reduces electron flow through the Q-junction and lowers Δ*Ψ*_mt_

**Fig. 1:**
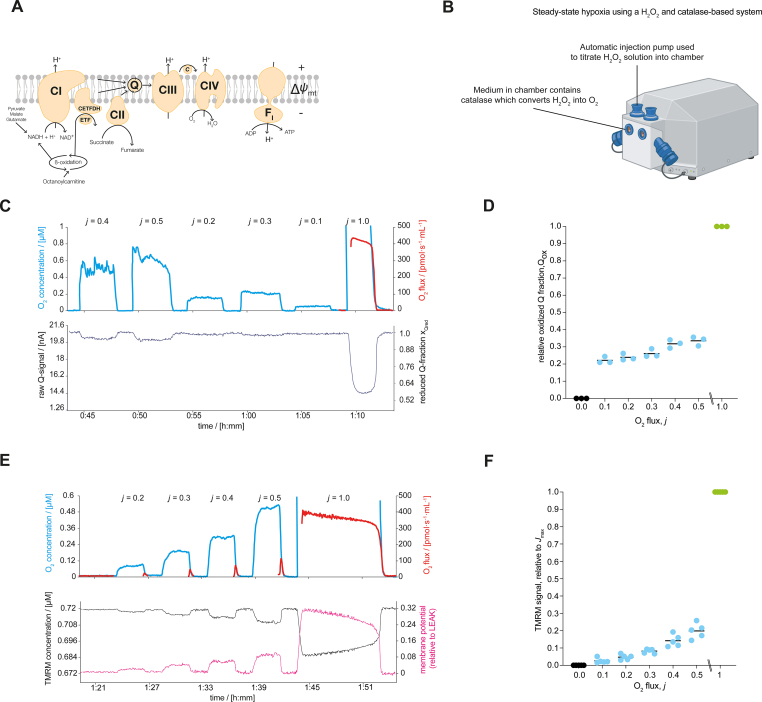
Functional hypoxia reduces the redox state of electron-transfer-reactive Q and lowers the mitochondrial membrane potential difference. A) Schematic representation of mitochondrial electron transfer system and coupling to oxidative phosphorylation (F_1_F_O_-ATPase). Electron transferring flavoprotein ETF is the redox carrier between β-oxidation and the respiratory Complex ETF dehydrogenase, CETFDH [29]. B) Schematic representation of the H_2_O_2_ and catalase-based system for steady-state respirometry used to study functional hypoxia. At steady-state the rate of H_2_O_2_ injection and conversion of H_2_O_2_ to O_2_ sets the mitochondrial metabolic flux *j*. C) Measurement of the redox state of the mitochondrial electron-transfer-reactive Q-pool (Q) under steady-states of hypoxia and at maximal O_2_ flux in isolated mitochondria from mouse cardiac muscle respiring on NADH-linked substrates, succinate, and fatty-acid in the presence of kinetically saturating ADP. D) Relative Q oxidation under anoxia (*j* = 0), functional hypoxia (*j* = 0.1 to 0.5) and at maximal O_2_ flux (*j* = 1) in mitochondria isolated from mouse cardiac muscle (*N* = 3). *j* is the ratio of the experimentally set *J*_O2_ to the measured OXPHOS capacity (*J*_max_). E) Mitochondrial membrane potential difference under steady states of hypoxia and at maximal O_2_ flux in mitochondria isolated from mouse cardiac muscle respiring on NADH-linked substrates, succinate, and fatty acid in the presence of kinetically saturating ADP. Increase in the TMRM concentration indicates a decrease in the mitochondrial membrane potential difference. F) Relative TMRM signal under anoxia (*j* = 0), functional hypoxia (*j* = 0.1 to 0.5) and at maximal O_2_ flux (*j* = 1) in mitochondria isolated from mouse cardiac muscle (*N* = 5). *j* is the ratio of the set *J*_O2_ to the measured OXPHOS capacity (*J*_max_ at kinetically saturating O_2_ concentrations). In panels D and F, the lines represent the means, dots are individual values.

In the first set of experiments, we investigated O_2_ control of mitochondrial electron transfer (Fig. 1A) in isolated mitochondria. The “apparent *K*_m_” or *p*_50_ of mitochondrial respiration – when oxygen partial pressure limits respiration to 50 % of the rate at kinetic oxygen saturation - ranges from 0.01 to 0.10 kPa (∼0.1 to 1 μM O_2_ or 0.08 to 0.8 mmHg) [14]. Therefore, to study O_2_ control of other mitochondrial parameters requires high resolution in the low μM range for O_2_ and sufficient time for the resolution of additional parameters being measured simultaneously [24]. To overcome this challenge, we used a steady-state high-resolution respirometry approach (Fig. 1B and S1) [24]. This allowed us to study functional hypoxia by setting mitochondrial respiration at specific O_2_-limited fractions of maximal O_2_ flux (*J*_max_; Fig. 1B and S1) through the titration of a hydrogen peroxide (H_2_O_2_) solution in the presence of excess catalase in the incubation medium (Fig. 1B and S1). At steady-state oxygen concentrations the rate of H_2_O_2_ injection and conversion of H_2_O_2_ to O_2_ sets the mitochondrial metabolic flux *j*. Maintaining a constant injection flow over a period (typically 3-6 min) allowed us to measure key mitochondrial parameters and functions of interest under steady states of hypoxia (Fig. 1B and S1). Importantly, there is agreement in the O_2_ kinetics observed in aerobic-anaerobic transitions and steady-states [24] making this approach the gold-standard (accurate steady state measurement of O_2_ levels with resolution better than ±1 nM O_2_ [13]; see Methods for further details) to study O_2_ control of mitochondrial electron transfer.

Providing substrates to support convergent electron entry into the Q-junction from oxidation pathways linked to NADH (via Complex I CI), succinate (via Complex II CII) and fatty acid (via electron transferring flavoprotein dehydrogenase Complex [29]) in the presence of saturating ADP concentrations (Fig. 1A) we first measured the redox state of the electron-transfer-reactive coenzyme Q (Q). Using the Q-mimetic coenzyme Q_2_ and a three-electrode system [30], at steady-state levels of hypoxia (limiting respiration to 0.1 to 0.5 *J*_max_), Q was more reduced compared with kinetically saturating O_2_ levels in mitochondria isolated from mouse heart (Fig. 1C, D, S2B) and brain (Figs. S2D and E).

To assess the consequence of oxygen-limited respiration on Δ*Ψ*_mt_ we applied steady-state respirometry using tetramethylrhodamine methyl ester (TMRM) in quench mode (Fig. 1E). Tracking the mt-membrane potential difference across steady states of hypoxia up to kinetically saturating oxygen concentrations revealed that oxygen limitation leads to a reversible depolarization of Δ*Ψ*_mt_ (indicated by increases in the measured TMRM concentration) in mitochondria isolated from mouse heart (Fig. 1E and F). In summary, oxygen limitation of respiration reduces electron flow and oxidative phosphorylation, and lowers the mitochondrial membrane potential difference.

### 2.2 Functional hypoxia reduces mitochondrial Ca^2+^ uptake

One important function of Δ*Ψ*_mt_ is to provide the driving force for Ca^2+^ uptake [31]. Mitochondrial Ca^2+^ influx occurs through the voltage-dependent anion channel (VDAC) on the outer mitochondrial membrane and through the highly selective mitochondrial calcium uniporter (MCU) across the mitochondrial inner membrane [28]. MCU function relies almost completely on two parameters: [Ca^2+^] in the area surrounding the channel and Δ*Ψ*_mt_ [28]. Therefore, we hypothesized that functional hypoxia would reduce mitochondrial Ca^2+^ uptake due to its effect on Δ*Ψ*_mt_.

We investigated mitochondrial Ca^2+^ uptake under functional hypoxia in mitochondria isolated from mouse heart using calcium green [32]. Given that Ca^2+^ can alter the activity of the mitochondrial electron transfer system [33,34] we first determined the conditions under which O_2_ flux was not affected by Ca^2+^. This was a necessary step as our aim was to compare mitochondrial Ca^2+^ uptake at maximal flux (*J*_max_) with Ca^2+^ uptake at a specific reduced fraction of *J*_max_. Any changes in flux due to Ca^2+^ during the measurement period would complicate the interpretation of our results. We therefore performed a sequential stepwise titration of CaCl_2_ (10 μM) whilst monitoring mitochondrial Ca^2+^ uptake simultaneously with O_2_ consumption (Fig. S3A). Mitochondrial Ca^2+^ uptake was stimulated starting at 10 μM CaCl_2_, however, at higher concentrations (starting at 30 μM) mitochondrial respiration was inhibited (Fig. S3A). We therefore tested if a single 20 μM titration of CaCl_2_ at saturating O_2_ concentrations (at *J*_max_) affected respiration. These experiments showed mitochondrial O_2_ flux to be stable for >6 min (Figs. S3B and C).

**Fig. 2:**
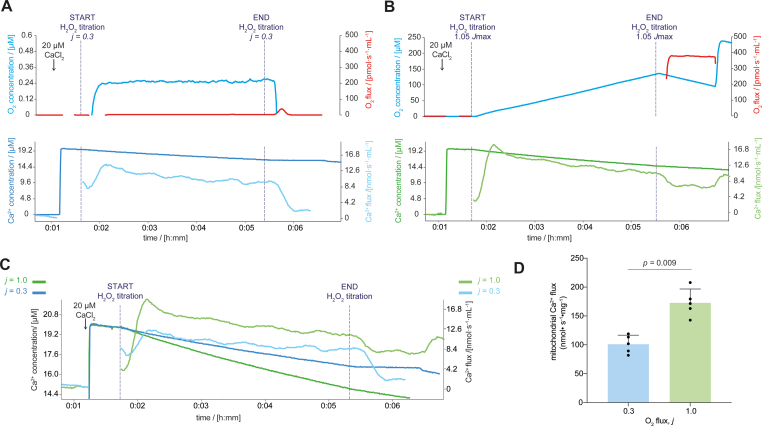
Functional hypoxia reduced mitochondrial Ca^2+^ flux in mitochondria isolated from mouse cardiac muscle. A) Mitochondrial Ca^2+^ flux under steady-state hypoxia. B) Mitochondrial Ca^2+^ flux at maximal O_2_ flux. C) Representative traces of extramitochondrial Ca^2+^ concentration and mitochondrial Ca^2+^ flux at maximal O_2_ flux (*J*_max_, green line) and under functional hypoxia (*j* = 0.3, blue line; superimposed from panels A and B). D) Mitochondrial Ca^2+^ flux under functional hypoxia (*j* = 0.3) and at maximal O_2_ flux (*j* = 1.0; *N* = 5) measured during the final 3 min of the 4-min H_2_O_2_ titration. In panel D the bars represent the means, error bars the standard deviations, dots are individual values.

Based on the preceding test results, 20 μM CaCl_2_ was applied to compare mitochondrial respiration at 0.3 *J*_max_ (*j* = 0.3; Fig. 2A) and at *J*_max_ (*j* = 1.0; Fig. 2B) for 4 min. Mitochondrial Ca^2+^ flux was lower when respiration was limited by O_2_ levels, i.e. in functional hypoxia (Fig. 2C and D). These data are in keeping with the reduced Δ*Ψ*_mt_ as the driving force for Ca^2+^ uptake under hypoxia (Fig. 1E and F). Taken together, our findings indicate that oxygen limitation of oxidative phosphorylation lowered Δ*Ψ*_mt_ and in turn reduced mitochondrial Ca^2+^ flux.

### 2.3 Myotubes reduce mitochondrial Ca^2+^ uptake under functional hypoxia

The ability of mitochondrial Ca^2+^ uptake to shape cellular events varies by cell type [28]. One cellular process where this plays a key role is muscle contraction [35,36]. In muscle tissue, mitochondrial Ca^2+^ influences not only energy metabolism during contraction [33] but also signalling for adaptive processes such as remodeling of mitochondrial metabolism [37]. Because of this important dual role of mitochondrial Ca^2+^ in muscle physiology, we chose to explore hypoxia further in skeletal muscle tissue.

**Fig. 3:**
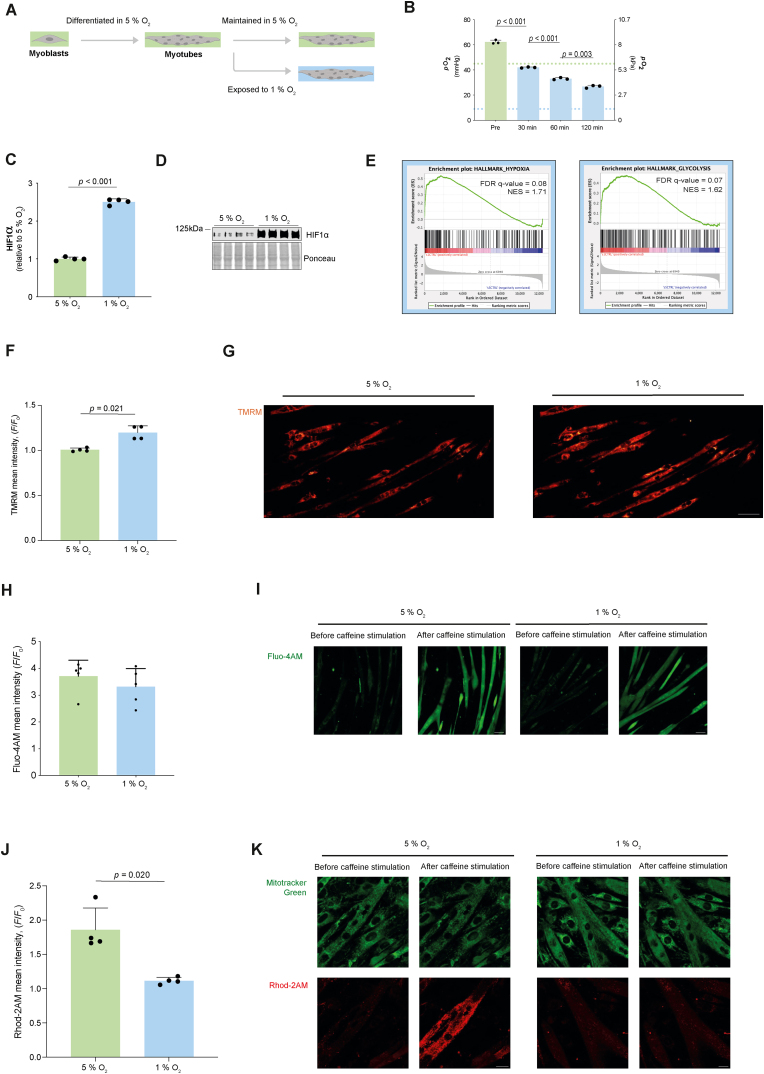
Mitochondrial Ca^2+^ uptake was lower under functional hypoxia in mouse myotubes. A) Culture conditions. B) Partial pressure of O_2_ in culture media upon exposure to 1 % O_2_ in the gas phase (*N* = 3). C) HIF1α protein content in myotubes 120 min after exposure to 1 % O_2_ in the gas phase (*N* = 4). Representative blot of HIF1α with ponceau (loading) stain in myotubes 120 min after exposure to 1 % O_2_ in the gas phase (*N* = 4). E) Hypoxia and glycolysis hallmark gene sets enriched in myotubes 120 min after exposure to 1 % O_2_ compared with 5 % O_2_ in the gas phase (*N* = 6). False discovery rate FDR. Normalized enrichment score NES. F) TMRM mean intensity under 5 % (*t* = 0 min) and after 120 min of exposure to 1 % O_2_ in the gas phase (*t* = 120 min; *N* = 4) in C2C12 myotubes. G) Representative images of C2C12 myotubes stained with TMRM exposed to 5 % or 120 min after exposure to 1 % O_2_ in the gas phase. Scale bar is 50 μm. An increase in the TMRM intensity indicates a decrease in the mitochondrial membrane potential difference. H) Quantification of Fluo-4AM mean intensity in response to caffeine stimulation in myotubes 120 min after exposure to 5 % or 1 % O_2_ in the gas phase (*N* = 5). I) Representative images of C2C12 myotubes stained with Fluo-4AM, exposed to 5 % or 1 % O_2_ and stimulated with caffeine. Scale bar is 50 μm. J) Response of Rhod-2AM fluorescence to caffeine stimulation in C2C12 myotubes 120 min after exposure to 5 % or 1 % O_2_ in the gas phase (*N* = 4). K) Representative images of C2C12 myotubes stained with Mitotracker Green and Rhod-2AM, exposed to 5 % or 1 % O_2_ and stimulated with caffeine. Scale bar is 20 μm. In panels B, C, F, H and J, the bars represent the means, error bars the standard deviations, dots are individual values.

Having found that functional hypoxia impacts Ca^2+^ flux in mitochondria isolated from mouse heart, we next considered whether hypoxia also exerts control over mitochondrial Ca^2+^ uptake in living cells. To explore this hypothesis, we first performed experiments to assess the culture conditions required to model functional hypoxia in myotubes (Fig. 3A). In most published papers using cellular models, ambient air humidified to 90 % and enriched with 5 % carbon dioxide is used, which represents an oxygen pressure (*p*_O2_ ∼20 kPa at sea-level, ∼18.5 % O_2_) that is higher than that experienced by most tissues *in situ* [20]. There is a growing appreciation that the oxygen levels used in cell culture (with primary cells and cell lines) profoundly affect the observed biological reproducibility and *in vivo* relevance [20]. Given the importance of culturing cells under physiological O_2_ levels to improve *in vivo* relevance we chose to use 5 % O_2_ to mimic normoxic *in vivo* skeletal muscle O_2_ pressure (∼4-8 % O_2_ at an atmospheric pressure ∼100 kPa [11]). Evidence from human skeletal muscle suggests that under ambient hypoxia (fraction of inspired O_2_ = 10 %) *in vivo*, resting skeletal muscle O_2_ pressures are ∼1-3 % O_2_ [11].

We exposed differentiated myotubes cultured under 5 % O_2_ acutely to 1 % O_2_ in the gas phase and used an optical probe to measure the partial pressure of oxygen (*p*_O2_) in the media. A 2 h exposure was necessary to reduce media *p*_O2_ from normoxia to physiological values known to occur in acute ambient hypoxia in humans ([8]; Fig. 3B). At this timepoint we assessed if O_2_ in the intracellular environment was also decreased by measuring the protein content of the transcription factor HIF1α (which increases exponentially from 5 % to 1 % O_2_ see Ref. [38]) by western blot and performing bulk RNA-sequencing to quantify the expression of gene-sets involved in the cellular response to hypoxia. HIF1α protein (Fig. 3C-D) and the “Hypoxia” and “Glycolysis” hallmark gene-sets (Fig. 3E, Dataset S1) were among those enriched in myotubes exposed to 1 % compared with 5 % O_2_.

To test whether these conditions were functionally hypoxic we used confocal microscopy to perform live imaging on myotubes under 5 % and 1 % O_2_ to measure the Δ*Ψ*_mt_. Functional hypoxia decreased the Δ*Ψ*_mt_ (see data from isolated mitochondria in Fig. 1C and D). Indeed a 2 h exposure to 1 % increased the TMRM mean intensity indicating a decreased Δ*Ψ*_mt_ (Fig. 3F and G). As observed in isolated mitochondria this depolarization of Δ*Ψ*_mt_ was reversible and Δ*Ψ*_mt_ was rapidly restored by increasing O_2_ (Fig. S4A). Therefore, we used these culture conditions to assess the effect of functional hypoxia on Ca^2+^ dynamics in myotubes.

First, we examined if hypoxia affected Ca^2+^ release and myotube contraction. Using the cytosolic Ca^2+^ indicator Fluo-4AM and mitochondrial Ca^2+^ indicator Rhod-2AM we stimulated myotubes with 2.5 mM caffeine in the absence of extracellular Ca^2+^ to trigger Ca^2+^ release from the sarcoplasmic reticulum. There were no differences in cytosolic Ca^2+^ in response to caffeine stimulation under 5 % or 1 % O_2_ (Fig. 3H and I). However, mitochondrial Ca^2+^ uptake was lower in response to caffeine stimulation under 1 % compared with 5 % O_2_ (Fig. 3J and K) and occurred in the absence of changes of MCU protein content (Figs. S4B and C) although alterations in MCU activity due to other regulatory factors cannot be ruled out (e.g., mitochondrial calcium uptake protein (MICU); [39]). The conceptually simplest interpretation of these data is that in living cells mitochondrial Ca^2+^ uptake is reduced under hypoxia due to membrane depolarization.

### 2.4 Functional hypoxia alters mitochondrial adaptive responses to contractions in mouse myotubes and human skeletal muscle

**Fig. 4:**
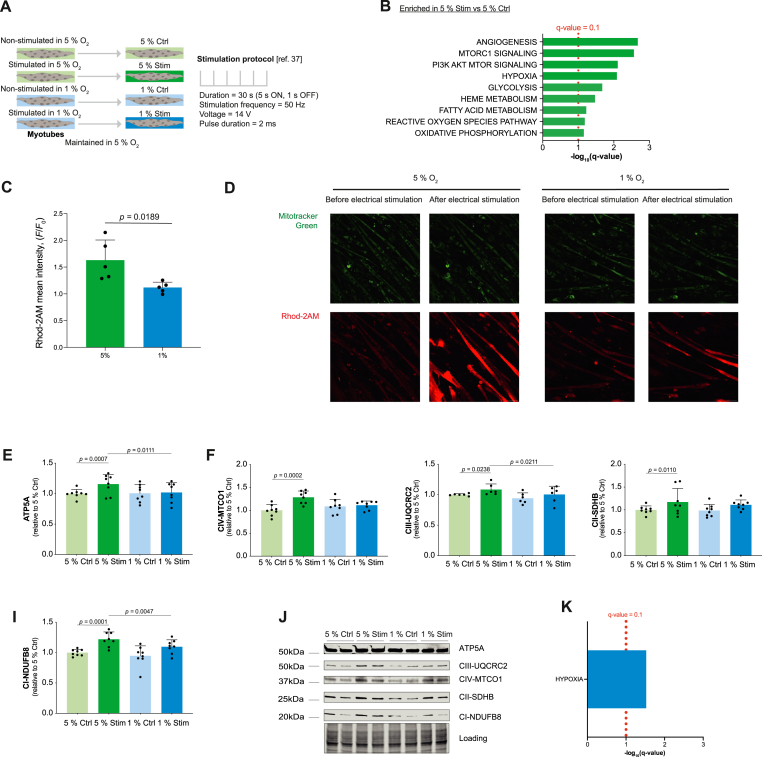
Functional hypoxia altered mitochondrial calcium uptake and mitochondrial responses to contractions in mouse myotubes. A) Culture conditions and electrical stimulation protocol. B) Hallmark gene sets enriched in myotubes immediately after stimulation in 5 % O_2_ compared with non-stimulated myotubes (*N* = 6). C) Response of Rhod-2AM fluorescence to electrical stimulation in C2C12 myotubes 120 min after exposure to 5 % or 1 % O_2_ in the gas phase (*N* = 4). D) Representative images of C2C12 myotubes stained with Mitotracker Green and Rhod-2AM, exposed to 5 % or 1 % O_2_ and electrically stimulated using the protocol shown in panel A. Images are taken immediately before- and after the electrical stimulation protocol. Scale bar is 50 μm. E-I) ATP5A (E), CIV-MTCO1 (F), CIII-UQCRC2 (G), CII-SDHB (H) and CI-NDUFB8 (I) protein content in C2C12 myotubes 48 h after stimulation (*N* = 7). J) Representative blot of ATP5A, CIII-UQCRC2, CIV-MTCO1, CII-SDHB and CI-NDUFB8 with loading total protein stain from C2C12 myotubes 48 h after stimulation. K) Hallmark gene sets enriched in myotubes immediately after stimulation in 1 % O_2_ compared with myotubes stimulated in 5 % O_2_ (*N* = 6). In panels B and K the bar represents the false discovery rate (FDR) for the specified group comparison. In panels C and E-I, the bars represent the means, error bars the standard deviations, dots are individual values.

Our data support a model whereby a reduction in Δ*Ψ*_mt_ under hypoxia decreases muscle mitochondrial Ca^2+^ uptake in response to Ca^2+^ release from the sarcoplasmic reticulum. This process is critical for the muscle response and metabolic remodeling upon muscle contraction including increased protein content of OXPHOS subunits and mitochondrial supercomplex formation [37]. Therefore, we decided to test whether these immediate changes under functional hypoxia could influence mitochondrial adaptions following a period of recovery in normoxia. To do so, we first performed experiments in electrically stimulated C2C12 myotubes (Fig. 4A). This electrical stimulation protocol induces Ca^2+^ release from the sarcoplasmic reticulum, myotube contractions, mitochondrial Ca^2+^ uptake (Videos S1, S2) and elicits mitochondrial adaptions under culture conditions with ∼18.5 % O_2_ [37].

Supplementary video related to this article can be found at https://doi.org/10.1016/j.redox.2024.103037

The following is/are the supplementary data related to this article:

Video S1 (separate file). Live imaging of cytosolic calcium in response to electrical stimulation in myotubes. Myotubes stained with the cytosolic Ca^2+^ indicator Fluo-4AM. Electrical pulses (voltage: 14 V, pulse duration: 2 ms) sent at 0.75 Hz for ∼35 s starting at ∼10 s. The frame rate is 3.79 frames per s. Images are taken in one focal plane and were recorded simultaneously with those shown in Video S2. Video S1

Video S2 (separate file). Live imaging of mitochondrial calcium in response to electrical stimulation in myotubes. Myotubes stained with the mitochondrial Ca^2+^ indicator Rhod-2AM. Electrical pulses (voltage: 14 V, pulse duration: 2 ms) sent at 0.75 Hz for ∼35 s starting at ∼10 s. The frame rate is 3.79 frames per s. Images are taken in one focal plane and were recorded simultaneously with those shown in Video S1. Video S2

Therefore, we first explored the response of mitochondria to six 30 s bouts of electrical stimulation at 50 Hz under 5 % O_2_ (Fig. 4A). Compared with non-stimulated controls, the electrical stimulation increased the transcripts of many gene sets that are hallmarks of the response to intense exercise including “Oxidative phosphorylation” (Fig. 4B), mitochondrial Ca^2+^ accumulation (Fig. 4C-D) and induced pyruvate dehydrogenase dephosphorylation (PDH; an indirect readout of mitochondrial Ca^2+^ uptake; Figs. S4E–F; [37]) immediately after stimulation. Moreover, electrical stimulation increased the protein content of OXPHOS subunits 48 hours later (Fig. 4E-J) as reported for myotubes in ∼18.5 % O_2_ [37], and similar to the response of human skeletal muscle to sprint interval training in normoxia [37].

Applying the same electrical stimulation protocol to myotubes in hypoxia led to an enrichment of transcripts in the “Hypoxia” gene set (Fig. 4K, Dataset S1) and altered lipid metabolism (as measured by mass-spectrometry see Methods; Figs. S4G–H, Dataset S2) but not glucose uptake (assessed using a glucose uptake assay see Methods; Fig. S4I) compared with stimulation in 5 % O_2_. Dihydroceramides which increase rapidly upon hypoxic exposure [40] were the only lipid-class significantly affected by hypoxia (Fig. S4H). Stimulation in 1 % O_2_ resulted in less mitochondrial Ca^2+^ accumulation (Fig. 4C-D) and blunted PDH dephosphorylation (Figs. S4E–F) compared with stimulation in 5 % O_2_, suggesting reduced mitochondrial Ca^2+^ uptake during contractions under hypoxia. Additionally, we observed that hypoxia blunted increases in oxidative phosphorylation protein complexes 48 hours after stimulation (Fig. 4E-J) possibly a result of reduced mitochondrial Ca^2+^ uptake during the contractions.

**Fig. 5:**
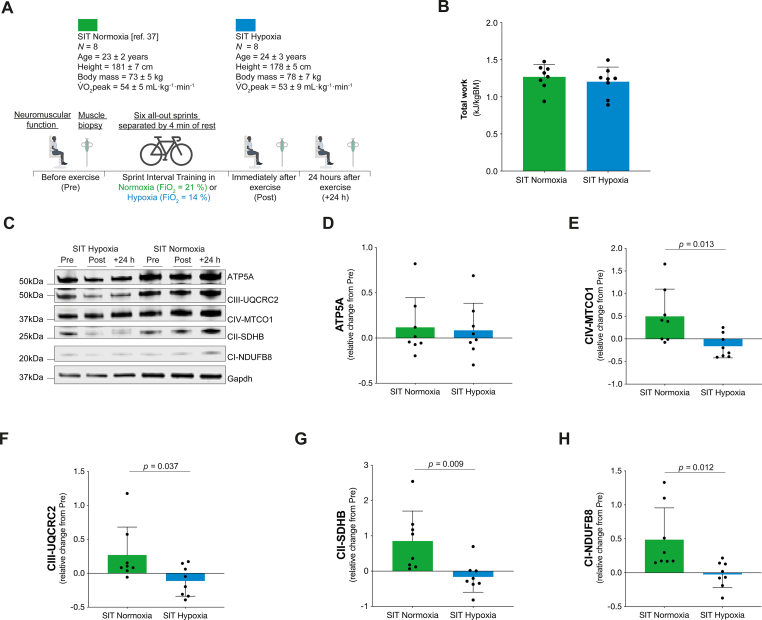
Functional hypoxia altered mitochondrial responses to contractions in human skeletal muscle. A) Human exercise study protocol. B) Total work performed (expressed relative to body mass) during the SIT session (*N* = 8). C) Representative blot of ATP5A, CIII-UQCRC2, CIV-MTCO1, CII-SDHB, CI-NDUFB8 and Gapdh (loading control) from human *vastus lateralis* biopsies. D-H) Relative changes in ATP5A (D), CIV-MTCO1 (E), CIII-UQCRC2 (F), CII-SDHB (G) and CI-NDUFB8 (H) protein content from Pre to +24 h in human *vastus lateralis* biopsies (*N* = 8). In panels B and D-H, the bars represent the means, error bars the standard deviations, dots are individual values.

Finally, to examine if hypoxia influenced adaptions to muscle contractions in humans, we collected data from a single session of sprint interval training (SIT) in hypoxia in eight healthy young male participants and compared these results with published data under normoxia (fraction of inspired O_2_ F_i_O_2_ = 20.9 %; [37]). During maximal exercise in ambient normoxia mitochondrial respiration in leg muscle is likely limited up to ∼5 % [7], therefore we chose to use a fraction of inspired O_2_ of 14 % to model functional hypoxia as this has been shown to limit both whole-body- and single leg-oxygen consumption during exercise to a greater extent than normoxia [9,[41], [42], [43], [44]]. The SIT hypoxia group underwent the same experimental protocol as the SIT normoxia group (Fig. 5A; see also [37]) with the exception that the F_i_O_2_ was reduced for the exercise session (5-min warm-up and six all-out 30 s sprints separated by 4-min of rest (Fig. 5A; see also [37]). The protocol included neuromuscular function assessments and *vastus lateralis* muscle biopsy collection before, immediately after, and 24 hours after the exercise session (Fig. 5A; [37]).

Performing the exercise in normobaric hypoxia reduced blood oxygen saturation (Figs. S5A–E) but did not affect exercise performance (Figs. S5F and G), work performed during the session (Fig. 5B), or the extent and etiology of knee extensor neuromuscular fatigue after the session (Fig. S5H-O, [37]). However, hypoxia blunted the previously reported [37] increases in respiratory Complexes CI, CII, CIII and CIV after exercise (Fig. 5C-H, [37]). These data suggest that the role of hypoxia on the adaptions of mitochondria to exercise training may be linked to reduced mitochondrial Ca^2+^ uptake.

## 3 Discussion

Mitochondrial respiration extends beyond ATP generation – with the organelle participating in many cellular and physiological processes [15]. How changes in respiration under functional hypoxia are relayed by the electron transfer system to elicit mitochondrial adaption to hypoxic exposure remain poorly defined. Studies with isolated mitochondria describing parallel changes in redox components of the mitochondrial electron transfer system with respiration [24] render it an appropriate hub for coordinating cellular adaption to changes in oxygen levels. Advancements in understanding the mechanisms through which hypoxia regulates biological outcomes has proved challenging, largely, due to the difficulty to integrate studies of isolated mitochondria under well-controlled and defined O_2_ levels to hypoxia in intact humans. Here we performed experiments under conditions of functional hypoxia (i.e., when O_2_ levels limit mitochondrial respiration; [5]) in carefully chosen and relevant models: isolated mitochondria, myotubes and exercising humans. Our findings in isolated mitochondria and myotubes suggest that O_2_ control of mitochondrial oxidative phosphorylation can relay changes in respiration and reduce mitochondrial Ca^2+^ uptake. We propose that alteration of this mitochondrial Ca^2+^ signal may influence how muscle mitochondria adapt after contractions in conditions of functional hypoxia.

Historically, addressing questions under conditions of functional hypoxia has been challenging due to the low μM O_2_ conditions required to limit mitochondrial respiration [5,6,24]. As an example, some previous studies [45,46] have investigated *“hypoxia”* albeit using effectively “hyperoxic” conditions where there is no oxygen limitation of mitochondrial respiration (i.e., “functionally normoxic”) [5]. Comparing control (200 μM O_2_) and *“hypoxic”* conditions (50 μM O_2_) in isolated mitochondria from rat hearts revealed no changes in Δ*Ψ*_mt_ or mitochondrial Ca^2+^ [45] in contrast to the results reported here showing lowered Δ*Ψ*_mt_ (Fig. 1E and F) and reduced mitochondrial Ca^2+^ flux (Fig. 2) under functional hypoxia (mitochondrial respiration at specific O_2_-limited fractions of maximal O_2_ flux). Only one study [47] has previously investigated mitochondrial Ca^2+^ uptake under low-oxygen levels. In rat cardiomyocytes electrically stimulated under *anoxia* (*p*_O2_ limit of detection <0.003 kPa; [48]) mitochondrial Ca^2+^ was not increased when the cells entered a state of rigor after 30-40 min [47]. We did not specifically investigate mitochondrial Ca^2+^ flux under anoxia, but we did not detect Ca^2+^ uptake after titration of CaCl_2_ under anoxia (Fig. 2A-C). Although it is very well established under anoxia that collapse or decline of the protonmotive force can have drastic consequences on mitochondrial ATP generation, here we went beyond this and quantitatively studied partial oxygen limitation of respiration using a method to set oxygen flux. Increasing O_2_ levels above anoxia, we then detected mitochondrial Ca^2+^ flux (Fig. 2). This flux was reduced by ∼35 % in isolated mitochondria when mitochondrial respiration was oxygen-limited at 0.3 relative to *J*_max_. In myotubes, mitochondrial Ca^2+^ accumulation was also reduced under hypoxia in response to sarcoplasmic reticulum Ca^2+^ release (Fig. 3J and K). The observed reduction in mitochondrial Ca^2+^ flux of ∼35 % in response to 20 μM CaCl_2_ in isolated mitochondria would equate to a reduction in Δ*Ψ*_mt_ from 160 to ∼110 mV (see Fig. 1c in Ref. [31]).

Although we did not use a method that allowed us to calculate Δ*Ψ*_mt_ in terms of absolute transmembrane potential differences ([mV]; e.g., Ref. [49]), we did obtain information on changes in Δ*Ψ*_mt_ under steady states of hypoxia in isolated mitochondria (Fig. 1E and F) and myotubes (Fig. 3F and G). We found that under hypoxia there was a depolarization of the Δ*Ψ*_mt_ (Figs. 1F and 3F). The relative changes in TMRM signal under hypoxia compared with kinetically saturating oxygen levels in mitochondria isolated from mouse heart and 5 % O_2_ in skeletal muscle myotubes were 5- and 1.25-fold, respectively. Although we cannot provide an explanation for this difference it is likely not due to an altered plasma membrane potential difference which is unaffected even after 30 min of *anoxia* (<1 nM O_2_ combined with lactate efflux inhibition) in rat cardiomyocytes [50]. The depolarization of Δ*Ψ*_mt_ (relative change in TMRM signal) in myotubes after 2 h of exposure to 1 % O_2_ (∼10 mmHg or 1.3 kPa) in the gas phase is less than that reported after 40 minutes of *ischemia* (measured *p*_O2_ < 2 mmHg or 0.3 kPa) in rat cardiomyocytes [12]. Taken together, these results support the notion that hypoxia reduces mitochondrial Ca^2+^ uptake via Δ*Ψ*_mt_.

The change in Δ*Ψ*_mt_ under functional hypoxia in isolated mitochondria occurred with a ∼4-fold greater reduction of the Q-pool compared with functional hypoxia (Fig. 1C and D). This result is consistent with a pronounced reduction of the cytochromes *aa*_3_, *b* and *c* under steady-state hypoxia [24], and an increased NAD(P)H reduction after 40 min of ischemia in Chouchani et al. [12].

On the other hand, the ∼4-fold greater reduction of Q under functional hypoxia compared with functional normoxia contrasts with previous reports. Using mass-spectrometry to measure coenzyme Q reduction from mouse hearts after 30-min *ischemia* there were no changes in coenzyme Q redox state [51] whereas there was a ∼1.5- to 2-fold increase in reduction of Q in cardiomyocytes [50]. These discrepancies most likely arise from the different methods (electrochemical Q-sensor versus Q-extraction method) and preparations (isolated mitochondria or whole tissue/cells). Whereas these fold changes in isolated mitochondria and cells do not necessarily align, there does seem to be a current consensus in the literature cited above that under functional hypoxia the electron transfer system is more reduced. Numerous reports have unraveled the role of metabolites and reactive oxygen species in relaying this information to influence cellular adaption. Here, we suggest that Ca^2+^ may be the relay for altered mitochondrial adaption to muscle contraction under hypoxia.

We observed that hypoxia blunted the increase in protein content of OXPHOS Complexes after muscle contractions (Fig. 4E-J, 5C-H). Indeed, direct measurements of mitochondrial Ca^2+^, an important signal for cellular adaption, after contractions showed reduced mitochondrial Ca^2+^ accumulation under hypoxia (Fig. 4C-D). Further supporting the notion that Ca^2+^ may be the relay for altered adaption, the response of OXPHOS proteins observed in the present study is similar to what was observed in response to contractions in myotubes with reduced MCU protein content [37]. Whether this alteration in mitochondrial Ca^2+^ equilibrium is due to changes in influx [28,39] or efflux [52] cannot be fully determined. But our data showing that under experimental conditions of increased extramitochondrial Ca^2+^ there is a blunted increase in free mitochondrial Ca^2+^, suggesting that it is reduced mitochondrial Ca^2+^ uptake due to a lower Δ*Ψ*_mt_ that leads to less free mitochondrial Ca^2+^.

Logically it is tempting to suggest that targeting the MCU to increase mitochondrial Ca^2+^ uptake under hypoxia might recover the blunted response of OXPHOS proteins after a period of recovery. However, mitochondrial Ca^2+^ can stimulate or inhibit mitochondrial respiration depending on the concentration in the matrix [34], and Ca^2+^ influences the mt-membrane potential difference [53]. Stimulating respiration under hypoxia could drive the cells towards “deeper” hypoxia and increasing matrix Ca^2+^ concentration could potentially collapse Δ*Ψ*_mt_, halting oxidative phosphorylation [54]. Therefore, the logic of simply increasing mitochondrial Ca^2+^ uptake under hypoxia to improve the response of OXPHOS proteins after a single session of exercise may not be so straightforward, and other studies suggest this may not be necessary. For example, chronic exercise training studies comparing training in hypoxia with normoxia show that the changes in whole-body *V*_O2_ and muscle mitochondrial respiration are similar after several weeks [55].

Although our data suggest a role for reduced mitochondrial Ca^2+^ uptake in the response of muscle mitochondria to contractions under hypoxia, other factors contribute. For example, hypoxia has been shown to impair muscle protein synthesis [56] and induce signalling via other pathways e.g., the HIF pathway [38] which was activated in myotubes (Fig. 3, Fig. 4K). It would seem logical that when exposed to hypoxia the system activates pathways to promote adaption of other metabolic pathways e.g., glycolysis (Fig. 3E) at the expense of mitochondrial oxidative phosphorylation [57,58]. But how exactly could a reduced mitochondrial Ca^2+^ flux contribute to this? One interesting avenue not explored in this manuscript is the effect of the different mitochondrial Ca^2+^ fluxes and changes in free mitochondrial Ca^2+^ during and after contractions in functional normoxia and hypoxia on Δ*Ψ*_mt_. This and changes to total mitochondrial Ca^2+^ could potentially have important consequences for signaling events (e.g., mitochondrial reactive oxygen species production [26,52], the import of proteins into mitochondria [27]) that coordinate cellular remodeling and warrant further investigation. Regarding extrapolation of our data in the context of adaptation to hypoxia, despite paying careful attention to O_2_ levels and using the best available methodologies, there are numerous other changes under hypoxia (e.g., pH, CO_2_) that we did not control. We hope that illustrating how oxygen uses the mitochondrial electron transfer system as a hub for coordinating cellular adaption will stimulate more research on the role of mitochondria in human adaptation to hypoxia including high-altitude and hypoxia-related diseases (e.g., chronic obstructive pulmonary disease, sleep apnea [59]).

## 4 Conclusion

We propose that under functional hypoxia with limitation of electron flow through the Q-junction (bottleneck effect), there is a progressive reduction of the electron-transfer-reactive Q-pool and a concomitant partial depolarization of Δ*Ψ*_mt_. In turn this leads to a reduction in mitochondrial Ca^2+^ uptake during muscle contractions through which hypoxia then influences mitochondrial adaptive remodeling to exercise.

## 5 Materials and methods

### 5.1 Key resource table

**Table:** 

REAGENT or RESOURCE	SOURCE	IDENTIFIER
**Antibodies**		
Gapdh anti-mouse	Abcam	Cat#8245
HIF1α anti-rabbit	Cayman Chemicals	Cat#10006421
MCU anti-rabbit	Sigma-Aldrich	Cat#016480
Total OXPHOS anti-mouse	Abcam	Cat#110413
PDH phospho S293 anti-mouse	Abcam	Cat#92696
Donkey anti-rabbit IgG polyclonal antibody	LI-COR	Cat#926-32213
Donkey anti-mouse IgG polyclonal antibody	LI-COR	Cat#926-32212
Donkey anti-mouse IgG polyclonal antibody	LI-COR	Cat#926-68022
Donkey anti-rabbit IgG polyclonal antibody	LI-COR	Cat#926-68023
**Chemicals, Peptides and Recombinant Proteins**		
DMEM	Thermo Fisher Scientific	Cat#41966-052
Fetal bovine serum	Thermo Fisher Scientific	Cat#1008247
Penicillin streptomycin	Thermo Fisher Scientific	Cat#15140122
Non-essential amino acids	Thermo Fisher Scientific	Cat#11140035
Horse serum	Thermo Fisher Scientific	Cat#26050088
Trypsin-EDTA (0.05 %) phenol red	Thermo Fisher Scientific	Cat#25300054
Trypsin	Sigma-Aldrich	Cat#T0303
Dimethyl sulfoxide	Sigma-Aldrich	Cat#8418
Paraformaldehyde	Thermo Fisher Scientific	Cat#J19943.K2
Fluo-4AM	Thermo Fisher Scientific	Cat#F142010
Mitotracker Green	Thermo Fisher Scientific	Cat#M7514
Rhod-2AM	Thermo Fisher Scientific	Cat#R1245MP
TMRM	Thermo Fisher Scientific	Cat#T668
MiR05-Kit	Oroboros Instruments	Cat#60101-01
Dithionite	Sigma-Aldrich	Cat#71669
Catalase	Sigma-Aldrich	Cat#9322
Hydrogen peroxide H_2_O_2_	Life Technologies	Cat#A36006
Calcium chloride CaCl_2_	Sigma-Aldrich	Cat#21115
Pyruvate	Sigma-Aldrich	Cat#P2256
Malate	Sigma-Aldrich	Cat#M1000
Glutamate	Sigma-Aldrich	Cat#G1626
Succinate	Sigma-Aldrich	Cat#2378
Cytochrome *c*	Sigma-Aldrich	Cat#C7752
Adenosine diphosphate	Merck	Cat#117105-1 GM
Carbonyl cyanide 4-(trifluoromethoxy)phenylhydrazone FCCP	Sigma-Aldrich	Cat#C2920
Rotenone	Sigma-Aldrich	Cat#R8875
Octanoylcarnitine	APExBIO Technology	Cat#B6371-50 mg
Antimycin A	Sigma-Aldrich	Cat#A8674
Coenzyme Q_2_	Sigma-Aldrich	Cat#C8081
Caffeine	Sigma-Aldrich	Cat#C0750
RNAeasy micro kit	QIAGEN	Cat#74004
Calcium green 5 N	Thermo Fisher Scientific	Cat#C3737
**Experimental Models: Organisms/Strains**		
C57BL/6J wild-type male and female mice	The Jackson Laboratories	Cat#000664
**Experimental Models: Cell lines**		
C2C12 cells	ATCC	Cat#Crl-1772
**Software and algorithms**		
Datlab 7	Oroboros Instruments	Cat#20700
Datlab 8	Oroboros Instruments	Cat#20700
Acqknowledge 4.2	BIOPAC	Cat#ACK100W
Zen software	Zeiss	https://www.zeiss.fr/microscopie/produits/microscope-software/zen.html
R	R	http://cran.r-project.org/
ImageJ		https://imagej.nih.gov/ij/
ImageStudio	LI-COR	https://www.licor.com/bio/image-studio/
Prism 8	GraphPad	https://www.graphpad.com/
Gene Set Enrichment Analysis Software	UC San Diego & The Broad Institute	https://www.gsea-msigdb.org/gsea/index.jsp
**Other**		
Incubator with O_2_ control	BINDER	Cat#6026
WiseStir homogenizer HS-30E	Wisd Lab Instruments	Cat#DH.WO01010
Rotina 380R	Hettich Lab	Cat#1701
Bike ergometer	Lode Excalibur Sport	Cat#839E
Quark gas analyser	COSMED	n/a
High-voltage constant current stimulator	Digitimer	Cat#DS7A
Isolated voltage stimulator	Digitimer	Cat#DG2A
1-cm diameter electrode	Kendall Meditrace 100	n/a
5-cm diameter electrode	Dermatrode	n/a
5- x 10-cm electrode	Compex	n/a
MP150 acquisition system	BIOPAC	n/a
Oxylite Pro	Oxford Optronix	n/a
C-Pace stimulator	IONOPTIX	n/a
Custom made insert with carbon electrodes	Mecanostrain	www.mecanostrain.ch
C-Dish	IONOPTIX	
Nanodrop 2000	Thermo Fisher Scientific	Cat#ND-2000
Stellaris 5	Leica	n/a
ViiA 7 Real-Time PCR System	Thermo Fisher Scientific	Cat#4453545
4–15 % Mini-PROTEAN TGX precast protein gels	Bio-Rad	Cat#4561084
Glucose-uptake glo assay	Promega	Cat#J1314
REVERT 700 total protein stain kit	LI-COR	Cat#926-11010
Intercept Blocking Buffer	LI-COR	Cat#927-70003
Uncoated 35‐mm‐diameter glass‐bottom dishes	MatTek	Cat#P35G-0-10-C
Poly-d-lysine coated 35‐mm‐diameter glass‐bottom dishes	MatTek	Cat#P35GC-0-10-C
Stellina Imaging System	Leica	https://www.leica-microsystems.com
6-well plates	Corning	Cat#CL3506
Cytation 3	BioTek	n/a
Bard Magnum biopsy instrument	Bard Radiography	n/a
RS400 heart rate monitor	Polar	n/a
8000Q2 Sensor	Nonin Medical Inc.	n/a
BCA protein assay kit	Thermo Fisher Scientific	Cat#23225
DC protein assay kit	Bio-Rad	Cat#5000111
Oxygraph-2k, O2k	Oroboros Instruments	Cat#10003-01
NextGen-O2k	Oroboros Instruments	Cat#10101-01

## 6 Resource availability

### 6.1 Lead contact

Chris Donnelly, chris.donnelly@unil.ch.

### 6.2 Materials availability

Materials and data generated from this study are available upon request to the Lead Contact.

### 6.3 Data and code availability

The published article includes all datasets generated and analyzed in this study.

RNA-seq data have been deposited to GEO under accession number GSE225927.

## 7 Experimental models and subject details

### 7.1 Isolation of mitochondria from mouse tissues

Experiments on isolated mitochondria from mouse tissues were performed using male and female wild-type C57BL/6J (14 ± 4 weeks of age; Jackson Laboratories) housed in a temperature-controlled (22 °C) room with a 12/12-h light/dark cycle. All procedures involving animals were performed in accordance with the Austrian Animal Experimentation Act in compliance with the European convention for the protection of vertebrate animals used for experimental and other scientific purposes. After cervical dislocation, the heart and the brain were removed and placed in ice-cold biopsy preservation solution (BIOPS; 2.77 mM Ca-EGTA, 7.23 mM K_2_EGTA, 5.77 mM ATP, 6.56 mM MgCl_2_, 20 mM taurine, 15 mM phosphocreatine, 20 mM imidazole, 0.5 mM dithiothreitol, 50 mM MES hydrate, pH 7.1). Mitochondria were isolated as previously described [24,30,60,61]. A glass/Teflon Potter-Elvehjem homogenizer (Wisd laboratory instruments) and centrifuge (Andreas Hettich GmbH & Co. KG) were used. All procedures were carried out in an ice bath or at 4 °C.

Mouse heart mitochondria were isolated following Komlódi et al. [30]. Briefly, wet mass of the whole heart was determined, washed with ice-cold BIOPS, and minced with scissors in ice-cold BIOPS (1 mL). The tissue was then digested for 2.5 min in 2 mL of isolation buffer CP1 (100 mM KCl, 50 mM MOPS, 5 mM MgSO_4_, 1 mM EGTA, 1 mM ATP; pH 7.4) containing trypsin (9 mg of 13000- 20000 U trypsin/1 g wet mass) with continuous stirring. Immediately after the 2.5 min, 2 mL of isolation buffer CP2 (CP1 buffer plus 0.2 % bovine serum albumin) was added. The homogenate was transferred into a pre-cooled glass/Teflon potter and homogenized at ∼1000 rpm (five strokes) in 2-mL isolation buffer CP1. The homogenate was transferred to a 50-mL Falcon tube containing 3 mL isolation buffer CP2 and centrifuged at 800 *g* for 10 min. Using a new 50-mL Falcon tube, the supernatant was centrifuged at 10 000 *g* for 10 min. The supernatant was discarded, the pellet was resuspended in isolation buffer CP1 (final volume 2 mL), and centrifuged at 10 000 *g* for 10 min. The supernatant was discarded, and the mitochondrial pellet was finally resuspended in 150 μL KME buffer (100 mM KCl, 50 mM MOPS, 0.5 mM EGTA).

Mouse brain mitochondria were isolated following Sumbalová et al. [62]. Briefly, wet mass was determined, and the tissue was cut into small particles with sharp scissors in isolation buffer C (320 mM sucrose, 10 mM Tris-Cl, 1 mM K-EDTA, 2.5 g/L fatty acid-free bovine serum albumin: pH 7.4). The medium was discarded, the tissue suspended in isolation buffer C (0.1 g tissue/1 mL), transferred to a pre-cooled glass/Teflon potter, and homogenized at 1000 rpm (five strokes). The homogenate was transferred to a 50-mL Falcon tube (0.5 g tissue/20 mL homogenate) and centrifuged at 1000 *g* for 10 min. The pellet was discarded, and the supernatant was centrifuged at 6200 *g* for 10 min. The supernatant was removed, the pellet resuspended in 0.5 g tissue/10 mL of isolation buffer D (320 mM sucrose, 10 mM Tris-Cl, 1 mM K-EDTA; pH 7.4) and centrifuged at 6200 *g* for 10 min. The supernatant was discarded, and the mitochondrial pellet was finally suspended in 500 μL isolation buffer D.

Each mitochondrial suspension was gently mixed with a 200-μL pipette (five up-down cycles), and 10 to 20 μL of heart or brain mitochondrial suspension was injected with a 50-μL Hamilton syringe into the O2k-chamber through the titration capillary of the stopper, respectively.

### 7.2 Cell lines

C2C12 mouse skeletal muscle myoblasts (American Type Culture Collection) were grown in proliferation medium composed of Dulbecco's modified Eagle's medium (DMEM; Thermo Fisher Scientific) supplemented with 10 % fetal bovine serum (Thermo Fisher Scientific), 100 IU/mL penicillin, 100 μg/mL streptomycin (Thermo Fisher Scientific) and 1 % non-essential amino acids (Thermo Fisher Scientific) and maintained at 37 °C in a humidified atmosphere with 5 % O_2_ and 5 % CO_2_ (Binder Incubators). To induce differentiation, myoblasts were grown to 80-90 % confluence and the proliferation medium was then replaced with a differentiation medium (pre-equilibrated at 5 % O_2_ and 5 % CO_2_) consisting of DMEM supplemented with 2 % horse serum (Thermo Fisher Scientific).

### 7.3 Human exercise study recruitment

Sixteen male participants were recruited for the study which was approved by the local ethics committee (protocol 2017-00303) and performed in accordance with the Helsinki declaration. Written informed consent was received from each participant prior to study inclusion. All participants were healthy and recreationally physically active. They were familiarized with the electrical stimulation and voluntary contraction procedures at least 48 h before the first experimental session. In this familiarization session participants also performed an incremental test to exhaustion on a cycle ergometer to determine $\dot{V}$_O2peak_ [37,63]. The participants were allocated to one of two sprint interval training (SIT) groups (SIT Normoxia and SIT hypoxia; *N* = 8 per group) based on their $\dot{V}$_O2peak_ to obtain two homogenous groups. Data from the SIT Normoxia group have been reported previously in Zanou et al. ([37]; the group named “SIT”).

## 8 Method details

### 8.1 Steady-state hypoxia experiments

Steady-state hypoxia experiments were performed using the methods described in Harrison et al. [24] and high-resolution respirometers (O2k or NextGen-O2k; Oroboros Instruments: limit of detection of oxygen flux at ±1 pmol O_2_ · s^-1^ · mL^-1^ [64]) with a Titration-Injection micropump (TIP2k) for maintaining constant O_2_ concentrations during simultaneous measurements of the redox state of the electron-transfer-reactive Q, Δ*Ψ*_mt_, or mitochondrial Ca^2+^ uptake (termed ‘d parameters’). O_2_ flux (the negative time-derivative of the O_2_ concentration) was calculated in real-time by the DatLab software. Corrections of O_2_ flux for instrumental background were based on instrumental quality control tests [4,30,65]. Experiments were performed under constant stirring (750 rpm) at 37 °C using a calibrated instrument and mitochondrial respiration medium (MiR06; 0.5 mM EGTA, 3 mM MgCl_2_, 60 mM lactobionic acid, 20 mM taurine, 10 mM KH_2_PO_4_, 20 mM HEPES, 110 mM sucrose, 1 g/L BSA, catalase 280 u/mL; pH 7.1) or calcium respiration medium (CaR; 70 mM KCl, 110 mM sucrose, 1 mM MgCl_2_, 10 mM KH_2_PO_4_, 20 mM HEPES, catalase 280 u/mL; pH 7.1). For experiments investigating free Ca^2+^, MiR06 could not be used as it contains a high concentration of EGTA. CaR was used instead.

Oxygen consumption and the associated parameter (Δ*Ψ*_mt_, Q-redox state or mitochondrial Ca^2+^ flux) were recorded following injection of isolated mitochondria into the chamber (residual endogenous respiration; *Ren*), after NADH-linked substrates ― (CI-linked) pyruvate (P, 5 mM), malate (M, 2 mM), and glutamate (G, 10 mM) ―, succinate (S, 10 mM; CII-linked), and octanoylcarnitine (Oct, 0.5 mM; fatty acid oxidation) were added (LEAK state) and after titration of ADP at kinetically saturating concentration (2.5 mM; OXPHOS capacity, *J*_*P*_) to reach a stable O_2_ consumption at kinetically saturating O_2_ concentrations (range 100 to 200 μM).

Mitochondrial respiration depleted the O_2_ concentration in the closed chamber (anoxia). Mitochondria were allowed to remain anoxic for ∼2 min to obtain an anoxic signal for zero calibration of the polarographic O_2_ sensor [4]. The TIP2k was then used to titrate a specific volume of H_2_O_2_ into the chamber. This critically important step in the protocol allowed us to calculate the increase in O_2_ concentration per volume of H_2_O_2_ solution titrated into the chamber (catalase in MiR06 dismutates H_2_O_2_ to O_2_ and H_2_O). This information was the basis to calculate the flow of H_2_O_2_ [μL/s] required to set mitochondrial respiration at target oxygen-limited O_2_ fluxes from *j* = 0.1 to 0.6 relative to maximal flux for each isolated mitochondria preparation in each chamber (see Supplementary File S3). At steady-state oxygen concentrations the rate of H_2_O_2_ injection and conversion of H_2_O_2_ to O_2_ sets the mitochondrial metabolic flux *j*. A steady state is difficult to achieve at *j >* 0.6 and was less relevant to our research question, therefore the range *j* = 0.1 to 0.6 was studied using steady-state respirometry. Mitochondrial respiration then returned the system to anoxia before the TIP2k was used to maintain a target oxygen-limited O_2_ flux (*J*_O2_) for 3 - 6 min during which time the steady-state O_2_ concentration (*c*_O2_) and an associated parameter were recorded. The injection was then terminated, allowing the system to reach an anoxic state for 2 min for recording bracketing zero O_2_ calibrations (increasing the resolution of oxygen measurements by two orders of magnitude [64]) and anoxic calibrations for the associated parameter. Sufficient H_2_O_2_ solution was titrated to increase the O_2_ concentration in the chamber so that the mitochondria were returned to kinetically saturating O_2_ concentrations (>20 μM; [24]). In some experiments, several successive steady-state cycles were carried out before and after reoxygenation. The ratio *j* = *J*/*J*_*P*_ was chosen in random sequence within the range *j* = 0.1 to 0.6 to avoid any systematic effects. This procedure was repeated for as long as the mitochondria recovered to stable post-anoxic O_2_ consumption levels that were not less than 0.9 of the initial *J*_*P*_. This loss of mitochondrial respiratory capacity is observed independent of H_2_O_2_ injections and is therefore not related to any oxidative damage induced by such injections [24]. Inhibition of respiration by oxidative stress is not observed in the presence of excess catalase activity even in cases when H_2_O_2_ injections are more frequently applied [66] and with higher step changes in O_2_ concentration [67]. Residual oxygen consumption (*Rox*) was determined after inhibition of CIII by antimycin A (2.5 mM). For representative traces of oxygen flux, the trace is only shown when a measurement is performed.

The Q-redox state was measured amperometrically using the a three-electrode system as a part of the Q-Module of the NextGen-O2k (Oroboros Instruments) and the Q-mimetic CoQ_2_ (1 μM; Sigma Aldrich) as described in Komlódi et al. [30]. The working electrode was poised at the oxidation potential of CoQ_2_ determined by cyclic voltammetry. The reduced Q fraction was calculated by calibrating the raw CoQ_2_ signal against 1) the fully oxidized CoQ_2_ signal (*Ren*) and 2) the fully reduced CoQ_2_ signal (anoxia) and expressed as a fraction of fully reduced CoQ_2_ (Figs. S2A and C; [30]). The equation reduced Q + oxidized Q = 1 was used to convert the reduced Q fraction to the oxidized Q fraction [30]. To express the oxidized Q fraction relative to the oxidized Q fraction at maximal O_2_ flux (*J*_max_), values were divided by the oxidized Q fraction at *J*_max_ (Fig. S2B).

Δ*Ψ*_mt_ was investigated using the fluorescent dye TMRM ([68,69]; 1 μM; quench mode). Fluorescence was measured with Green Smart Fluo-Sensors (Oroboros Instruments; 525 nm excitation LED). Filters for the LED and photodiode were selected for TMRM. The fluorescence signal was calibrated before each experiment with stepwise (0.2 μM) titrations of TMRM from 0 to 1 μM. The calibrated TMRM concentration was used in the experiment. For expression of the TMRM signal (i.e., Δ*Ψ*_mt_) relative to maximum and minimum values, the LEAK state and anoxia were used respectively (Fig. S2F). To express the relative TMRM signal to the relative TMRM signal at maximal O_2_ flux, values were divided by the relative TMRM signal at *J*_max_.

Calcium Green-5N (2 μM; which does not inhibit respiration; Thermo Fisher) was used to measure mitochondrial Ca^2+^ uptake in response to changes in free Ca^2+^ in the chamber ([32]). Fluorescence was measured with Blue Smart Fluo-Sensors (Oroboros Instruments; 465 nm excitation LED). Filters for the LED and photodiode were selected for Calcium Green. After titration of mitochondria, 15 μM EGTA was added to the chamber. CaCl_2_ (Sigma Aldrich) was titrated to the desired concentration from a 10 mM stock solution. Calibration of the fluorescence signal was performed during the experiment by two-point calibration. The fluorescence signal before (no CaCl_2_ added) and after the first CaCl_2_ titration were used. Mitochondrial Ca^2+^ flux was calculated as the negative time derivative of the Ca^2+^ concentration by the DatLab software and was normalized by mitochondrial protein mass.

### 8.2 Mitochondrial protein

Mitochondrial protein content was determined based on Lowry et al. [70], using the DC protein assay (Bio-Rad) following the manufacturer's instructions. The absorbance was measured at 620 nm in a Tecan Infinite TM F200 spectrophotometer (Tecan).

### 8.3 Acute myotube hypoxia experiments

Culture medium was replaced with 2 mL of pre-equilibrated medium (5 % O_2_) and myotubes were exposed to either 5 % O_2_ or 1 % O_2_ in the gas phase for 2 h before electrical stimulation. This duration was to allow for equilibration of the O_2_ pressure in the gas phase with the aqueous phase (assessed using an optical O_2_ sensor, see below for method; Fig. 3B) and to achieve intracellular hypoxia (evaluated as an increase in hypoxia-inducible factor 1α protein content, see below for method, compared with 5 % O_2_; Fig. 3C&D).

### 8.4 Measurement of media O_2_ partial pressure

Partial O_2_ pressure (*p*_O2_) in the culture media was monitored using optical fluorescence technology that allows real-time detection of *p*_O2_ (Oxylite Pro, Oxford Optronix). The tip of the sensor was placed at the bottom of the well (i.e., at the deepest point of the media).

### 8.5 Western blot

A lysis buffer containing 20 mM Tris/HCl (pH 6.8), 2 mM EDTA (pH 8), 137 mM NaCl, 10 % glycerol, 10 % Triton X-100, 10 mM glycero-phosphate, 1 mM KH_2_PO_4_, 1 mM PMSF, 1 mM NaVO_3_, 50 mM NaF, 10 mM NaPPi, and a protease inhibitor mixture (Roche) was used to resuspend myotube pellets (100 μL/well of a 6-well plate) or human muscle biopsies (100 μL/5 mg of tissue). The preparation was homogenized with pipette tips for cells or a potter for biopsies, incubated for 1 h at 4 °C then sonicated. Nuclei and debris were then removed by centrifugation at 10 000 *g* at 4 °C for 10 min. Protein concentration was determined using the BCA kit (Thermo Fisher Scientific). Ten to 20 μg of protein were incubated with laemmli sample buffer containing SDS and 2-mercapto-ethanol (Bio-Rad) for 3 min at 95 °C, electrophoresed for 1 h in 4-15 % SDS-precast gradient gels (Bio-Rad), and wet transferred for 1 h onto PVDF membranes. Membranes were stained with REVERT Total Protein Stain (LI-COR) and total protein bands were quantified using the Image Studio software v 5.2.5 (LI-COR). The stain was removed using a removal buffer (LI-COR) and the membranes were blocked for 1 h at room temperature with PBS-LI-COR blocking buffer (LI-COR). Blots were incubated overnight with rabbit anti-Gapdh (Abcam), mouse anti-total OXPHOS (Abcam), rabbit anti-pyruvate dehydrogenase E1α phospho serine 293 (Abcam), mouse anti-pyruvate dehydrogenase (Abcam) or rabbit anti-mitochondrial calcium uniporter (Sigma). All the primary antibodies were used at 1: 1000 dilution.

For HIF1α blots myotubes in one well of a six-well plate were washed once with PBS pre-equilibrated at the appropriate O_2_ pressure and collected in a hot (75 °C) lysis buffer containing laemmli then sonicated. The lysates were then processed as described above using an anti-rabbit HIF1α primary antibody (Cayman Chemicals).

Membranes were washed in PBS-buffered saline-Tween 20 (TBS-T) and incubated for 1 h at room temperature with 1:10 000 IRDye 680- conjugated donkey anti-mouse or rabbit IgG (LI-COR) and 1:5000 IRDye 800-conjugated donkey anti-mouse or rabbit IgG (LI-COR) in blocking buffer. Immunoreactive bands were visualized using infrared fluorescence (IR-Odyssey scanner, LI-COR). Band densities were quantified using Image Studio v 5.2.5 (LI-COR). The protein intensity signal was normalized to Gapdh (which was stable across samples and conditions) in human samples while the total protein staining (ponceau for HIF1α blots) was used to normalize protein content quantified in myotubes. For the human biopsy samples, protein quantifications were expressed as the relative change from Pre [change = (+24 h-Pre)/Pre]. The protein quantifications for cell samples were reported to that of 5 % CTRL cells.

### 8.6 RNA-sequencing

Six biological replicates per condition were used. Upon removal from the incubator, cells were rinsed with PBS pre-equilibrated at the appropriate O_2_ pressure, pelleted, and then stored at -80 °C. RNA extraction was performed using an RNAeasy kit (QIAGEN).

RNA quality was assessed on a Fragment Analyzer (Agilent Technologies) and all RNAs had a RQN above 9.3. RNA-seq libraries were prepared from 500 ng of total RNA with the Illumina TruSeq Stranded mRNA reagents (Illumina) using a unique dual indexing strategy, and following the official protocol automated on a Sciclone liquid handling robot (PerkinElmer). Libraries were quantified by a fluorometric method (QubIT, Life Technologies) and their quality assessed on a Fragment Analyzer (Agilent Technologies).

Cluster generation was performed with 1.95 nM of an equimolar pool from the resulting libraries using the Illumina HiSeq 3000/4000 SR Cluster Kit reagents and sequenced on the Illumina HiSeq 4000 using HiSeq 3000/4000 SBS Kit reagents for 150 cycles (single end). Sequencing data were demultiplexed using the bcl2fastq2 Conversion Software (version 2.20, Illumina).

### 8.7 Gene expression data analysis

Quality check of RNA sequencing reads was performed with FastQC v0.11.7 [71]. Reads were aligned to the reference mouse model GRCm38 using STAR v2.5.4b [72] and expression levels were quantified using RSEM v1.3.0 [73] with default parameters. ENSEMBL IDs were converted to MGI symbols using Biomart [74]. Differential expression analysis among the 4 groups (1 % CTRL, 5 % CTRL, 1 % STIM, 5 % STIM) was performed using Limma [75] and edgeR [76] following the best practices published in Law et al. [77]. DESeq2 [78] was used to generate normalized counts to provide as input for downstream Gene Set Enrichment Analysis (GSEA [79]). GSEA was performed with default parameters using Hallmark Gene Sets [80] and a false-discovery rate (FDR) cut-off at 0.1 (*q*-value 0.1).

### 8.8 Confocal microscopy

C2C12 myoblasts were plated on uncoated 35‐mm‐diameter glass‐bottom dishes (MatTek) and cultured as described above. At the indicated time of measurement, they were loaded with Mitotracker Green (200 nM; Thermo Fisher Scientific), TMRM (1 μM; used in quench mode; Thermo Fisher Scientific), Fluo-4AM (5 μM, Thermo Fisher Scientific) or Rhod-2AM (1 μM; Thermo Fisher Scientific) solubilized in a pre-equilibrated Krebs solution (135.5 mM NaCl, 1.2 mM MgCl_2_, 5.9 mM KCl, 11.5 mM glucose, 11.5 mM HEPES, 1.8 mM CaCl_2_, pH 7.3) for 15 min in the incubator at the 5 % O_2_. Cells were then rinsed twice with a Ca^2+^-free Krebs solution (135.5 mM NaCl, 1.2 mM MgCl_2_, 5.9 mM KCl, 11.5 mM glucose, 11.5 mM HEPES, 200 μM Na-EGTA, pH 7.3).

Images were acquired by z-stack acquisition using a confocal microscope (Stellaris 5, Leica). with APO 20x dry- or APO 63x/1.40 oil-objectives, with gas (CO_2_ = 5 %, O_2_ = 5 or 1 %) and temperature (37 °C) controlled using a stage-top incubator (Ibidi). For acute hypoxic exposure the same 2 h exposure was used.

Two fields of view were acquired per biological replicate. Image analysis was performed on myotubes using ImageJ Software; fixed thresholds for signal intensity were applied across experiments, and the mean intensity was measured at the appropriate time point.

In some experiments, myotubes were stimulated with 2.5 mM (final concentration) caffeine to trigger Ca^2+^ release from the sarcoplasmic reticulum. To monitor dynamic changes, peak fluorescence intensities (observed 1-5 s after caffeine titration; *F*) were normalized to pre-caffeine stimulation values (*F*_0_). To account for potential changes in mitochondrial mass, the Rhod-2AM signal was normalized to the Mitotracker Green signal.

### 8.9 Design and fabrication of electrode holder for microscopy

To perform live imaging with oxygen control and electrical stimulation, a bespoke electrode holder was designed and fabricated (Mechanostrain). This electrode holder was designed to be compatible with the petri dishes and the stage top incubator and microsopy equipment described in the previous section was well as biocompatible with cells. To keep the electrical impedance as low as possible, a gold-plated copper strip was inserted on the top face of electrodes. By this way a greater contact surface between graphite electrode and connecting wire was possible and on the final assembly the value measured from the plug to the lower face of the electrode was less than 1 Ω. All electrical parts were silicone coated for electrical insulation.

Once the design was completed (Fig. S4D) the computed aided design & machining software (Autodesk Inventor) computed the trajectories of cutting tools (CNC Milling machine: Roland MDX-540) into the plain materials. Polymethyl methacrylate (PMMA) was used for its properties of clear plastic with good optical properties, good machining and polishing ability. A subsequent polishing was performed for clear and easy to clean surfaces. The holder was then used (see below) to electrically stimulate myotubes via carbon electrodes and held in place by magnetic clamps (Fig. S4D).

### 8.10 Electrical stimulation of C2C12 myotubes

Well-differentiated C2C12 myotubes (5 to 7 days after differentiation) were electrically stimulated.

For microscopy experiments, C2C12 myotubes in uncoated glass bottom dishes containing 2 mL of Ca^2+^-free Krebs solution were electrically stimulated (IONOPTIX stimulator, Mechanostrain electrodes, Fig. 4D) at 14 V with a stimulation frequency of 0.75 Hz and a 2-ms pulse duration (Videos S1, S2) or for 6 x 30 s (5 s on, 1 s off) separated by 4 min at 14 V with a stimulation frequency of 50 Hz and a 2-ms pulse duration (SIT-mimicking stimulation; [37]).

To monitor the dynamic changes peak fluorescence intensities in response to electrical stimulation (observed immediately after 6 x 30 s stimulation; *F*) were normalized to pre-electrical stimulation values (*F*_0_). To account for potential changes in mitochondrial mass, the Rhod-2AM signal was normalized to the Mitotracker Green signal.

For the other experiments, C2C12 myotubes in 6-well plates (Corning, NY, USA) containing 2 mL of differentiation medium were electrically stimulated (IONOPTIX stimulator and electrodes) for 6 x 30 s (5 s on, 1 s Off) separated by 4 min at 14 V with a stimulation frequency of 50 Hz and a 2-ms pulse duration (SIT-mimicking stimulation; [37]). The differentiation medium was replaced before and after the electrical stimulation. The cells were harvested immediately upon removal from the incubator at the appropriate time point.

### 8.11 Sample preparation for lipidomics

Cells were removed from the incubator, washed once with PBS pre-equilibrated at the appropriate O_2_ pressure, then the plates snap frozen on liquid nitrogen.

### 8.12 Lipid extraction

Lipids from frozen skeletal muscle myotubes were extracted by the addition of 2-propanol. Following the scraping, cells were collected to lysis tubes (with 2-propanol) and homogenized by the addition of beads in Precellys tissue homogenizer (2 x 20 s). The homogenized lipid solution was then centrifuged (at 4 °C for 15 min at 15 000 rpm) and the supernatant was collected and evaporated to dryness (in SpeedVac, LabConco). Finally, the dry lipid extracts were resuspended in 2-propanol (volume normalized to the protein content) spiked with Splash mixture (8 %) of isotopically labeled lipid standards (Avanti Lipids) and transferred to LC-MS vials for injection.

### 8.13 Broad-scale targeted lipid analysis

Myotube extracts were analyzed by hydrophilic interaction liquid chromatography coupled to tandem mass spectrometry (HILIC - MS/MS) in both positive and negative ionization modes using a Q-TRAP 6500 LC-MS/MS system (Sciex Technologies; [81]). In both, positive and negative ionization mode, the chromatographic separation was carried out on an Acquity BEH Amide, 1.7 μm, 100 mm × 2.1 mm I.D. column (Waters). Mobile phase was composed of A = 10 mM ammonium acetate in Acetonitrile: H_2_O (95: 5) and B = 10 mM ammonium acetate in Acetonitrile: H_2_O (50:50). The linear gradient elution from 0.1 % to 20 % B was applied for 2 min, then from 20 % to 80 % B for 3 min, followed by 3 min of re-equilibration to the initial chromatographic conditions. The flow rate was 600 μL/min, column temperature 45 °C and sample injection volume 2 μL. Optimized ESI Ion Drive Turbo V source parameters were set as follows: Ion Spray (IS) voltage 5500 V in positive mode and -4500 V in negative mode, curtain gas 35 psi, nebulizer gas (GS1) 50 psi, auxiliary gas (GS2) 60 psi, source temperature 550 °C. Nitrogen was used as the nebulizer and collision gas. Optimized lipid class-dependent parameters were used for data acquisition in scheduled multiple reaction monitoring (MRM) mode. Raw LC-MS/MS data were processed using the MultiQuant Software (version 3.0.3, Sciex technologies). For each lipid species the peak area was reported based on its extracted ion chromatograms (EICs) for the monitored MRM transitions.

### 8.14 Quality control of lipidomics

Data quality assessment was performed using pooled quality control (QC) samples analyzed periodically (every ten samples) throughout the entire batch and in a dilution series at the beginning and at the end of the run. The obtained tables (containing peak areas of detected lipids across all samples) were exported to “R” software for signal intensity drift correction using the LOWESS/Spline algorithm followed by peak filtering [[82], [83], [84]]. Peaks with CV > 30 % across QC samples and correlation with dilution factor <0.65 were removed from further statistical analysis. Lipid annotations used are sphingomyelins (SM), ceramides (CER), dihydroceramides (DCER), lactosylceramides (LCER), hexosylceramides (HCER), monoacylglycerols (MAG), diacylglycerols (DAG), triacylglycerols (TAG), cholesterol esters (CE), phosphatidylinositols (PI), phosphatidylcholines (PC), phosphatidylglycerols (PG), phosphatidylethanolamines (PE), phosphatidylserines (PS), lysophospholipid analogues (LPC, LPG, LPE, LPI, LPS) and free fatty acids (FFA). Data were log10-transformed and normality was tested for each species with the Shapiro test. Differences among stimulation and oxygen levels were assessed with 2-way ANOVA.

### 8.15 Glucose uptake assay

On the day before the assay, the differentiation media was replaced with DMEM containing no serum. At the time of the assay, the culture medium was removed, and cells were washed with PBS pre-heated to 37 °C. Then, cells were incubated with DMEM containing 1 μM insulin (Promega) for 1 h under the appropriate O_2_ pressure. The solution was then removed, and the cells were incubated with freshly prepared 0.1 mM 2-deoxyglucose (Promega) for 30 min under the appropriate O_2_ pressure. The uptake process was stopped and neutralized, and luciferase activities were measured using a Glucose Uptake-Glo Assay kit (Promega) and a plate reader (BioTek). Glucose uptake was analyzed according to the manufacturer's instructions. Fold changes relative to 5 % Ctrl were calculated as [fold change = (value - 5 % CTRL)/5 % CTRL].

### 8.16 Human exercise study protocol

Before the experimental session the participants refrained from physical activity and caffeine consumption for 24 h and 12 h, respectively. The SIT session consisted of 6 x 30 s all-out cycling bouts with 0.7 N∙m/kg body mass resistance on a cycle ergometer, separated by 4 min rest between bouts. The SIT Normoxia group performed the exercise session in a normobaric chamber with a fraction of inspired O_2_ (F_i_O_2_) of 20.9 % and the SIT hypoxia group with an F_i_O_2_ of 14 % [9]. The sprints were preceded by a standard warm-up on the cycle ergometer (5 min at 100 W). Heart rate (Polar) and peripheral capillary O_2_ saturation (S_p_O_2_) was collected from the earlobe (Nonin Medical Inc) before the first and at the end of each sprint. Peak and mean power [W] and the rate of perceived exertion (Borg scale; 6 - 20) for each sprint, and the total work performed [kJ] were collected.

### 8.17 Neuromuscular function assessments

Knee extensor neuromuscular function of the right (dominant) leg was tested before (Pre), immediately (Post) and 24 h after (+24 h) exercise under ambient normoxia. The tests consisted of a 5 s maximal voluntary contraction (MVC) with a superimposed 100 Hz doublet (paired stimuli) evoked via supramaximal electrical stimulation of the femoral nerve (twitch interpolation technique [85]) followed by supramaximal stimulations of a relaxed muscle evoked at 2 s intervals: a doublet at 100 Hz, 10 Hz, and a single stimulus to obtain the compound muscle action potential (M-wave).

### 8.18 Electromyography

The electromyographic (EMG) activity of the right *vastus lateralis* was recorded with pairs of silver chloride (Ag/AgCl) circular surface electrodes (Kendall Meditrace 100) positioned lengthwise over the middle part of the muscle belly with an inter-electrode (center-to-center) distance of 2 cm according to SENIAM recommendations [86]. The reference electrode was placed over the patella. EMG signals were amplified (gain: 1000), filtered through a 10-500 Hz band-pass filter, and digitized at a sampling frequency of 2 kHz using an AD conversion system (BIOPAC).

### 8.19 Electrical nerve stimulation

A high-voltage (maximum 400 V) constant-current stimulator (DS7AH, Digitimer) was used to deliver single and paired electrical stimuli (pulse width 1 ms). The cathode (Dermatrode) and the anode (Compex) were placed over the femoral nerve at the femoral triangle level beneath the inguinal ligament and on the lower part of the gluteal fold opposite to the cathode, respectively. The optimal stimulation intensity was determined by increasing the current until maximal twitch and M-wave amplitude responses were obtained. This intensity was then increased by 20 % (i.e. supramaximal) and kept constant for all subsequent tests.

### 8.20 Force recordings

Voluntary and evoked forces developed by the knee extensors were recorded at 1 kHz using an isometric ergometer consisting of a custom-built chair equipped with a strain gauge (STS 250 kg, sensitivity 2.0 mV/V and 1.7 mV/N, SWJ, China). The strain gauge was attached to the chair on one end and securely strapped above the ankle with a custom-made mold. Participants were seated with a knee angle of 90° and a trunk-thigh angle of 100° (180° = full extension). Extraneous movements of the upper body were limited by two crossover shoulder harnesses and a belt across the lower abdomen. Participants received visual feedback of the torque they produced during the MVCs. Force and EMG data were stored and analyzed off-line with commercially available software (BIOPAC).

### 8.21 Electromyography and torque analyses

Single electrical stimulation pulses were used to measure the amplitude of the first peak of the M-wave before and after exercise. Isometric MVC force was considered as the peak force attained during the voluntary contraction performed at a given time point. The amplitudes of the 10 Hz and 100 Hz paired stimuli (PS10 and PS100) before and after exercise were quantified to assess contractile alterations after exercise and the PS10:PS100 was used as an indicator of low-frequency force depression [87]. PS100 were delivered superimposed on and immediately after MVCs to assess the voluntary activation level (VAL), which was used as an index of central fatigue and assessed as: VAL = (1 − (superimposed PS100 force × (force level at stimulation/MVC force)/potentiated PS100 force)) × 100 [85]. Rate of force development/relaxation were calculated by dividing the peak twitch force by the time interval from the onset of force development/relaxation to peak force/complete relaxation.

### 8.22 Muscle biopsies

Needle biopsies were taken from the left (non-dominant leg) *vastus lateralis* muscle before, ∼ 10 min and 24 h after exercise [88]. Briefly, after skin sterilization and local anesthesia, a 1 to 2 mm long skin cut was made with the tip of a scalpel. Needle biopsies were collected using an automatic biopsy device. A 14-gauge disposable trocar mounted in the device was inserted through the cut, perpendicular to the muscle fibers, until the fascia was pierced. Three samples (∼15 mg each) were collected from one puncture site at each time point. Muscle samples were immediately frozen in liquid nitrogen and stored at -80 °C until analysis.

## 9 Quantification and statistical analysis

Data analysis was performed in Excel, R, and Prism as described above. All data (unless otherwise noted) was presented as mean ± SD. Unless otherwise noted, *P* values were calculated using one-way ANOVA for multiple comparisons involving a single variable, a two-tailed Student's *t*-test for pairwise comparisons and a two-way ANOVA for multiple comparisons involving two-variables. Details of statistical analyses and *N* values are found in the Figure Legends. *N* indicates biological replicates or number of human participants for *in vivo* experiments. Where applicable, *p* values were adjusted for multiple hypothesis testing using the Benjamini-Hochberg procedure.

## Significance statement

Molecular oxygen is vital for mammalian cells, which have special sensing mechanisms to respond to changes in oxygen levels. Here we show that oxygen limitation of oxidative phosphorylation reduces the flow of electrons through the mitochondrial electron transfer system with a bottleneck effect at the coenzyme Q-junction. In skeletal muscle this lowers the mitochondrial membrane potential difference, which reduces mitochondrial calcium uptake. By altering this calcium signal, muscle mitochondria adapt their response to exercise under functional hypoxia.

## CRediT authorship contribution statement

**Chris Donnelly:** Conceptualization, Data curation, Formal analysis, Funding acquisition, Investigation, Methodology, Project administration, Resources, Supervision, Validation, Visualization, Writing – original draft, Writing – review & editing. **Timea Komlódi:** Data curation, Formal analysis, Investigation, Methodology, Writing – review & editing. **Cristiane Cecatto:** Data curation, Formal analysis, Investigation, Methodology, Writing – review & editing. **Luiza H.D. Cardoso:** Data curation, Formal analysis, Funding acquisition, Investigation, Methodology, Writing – review & editing. **Anne-Claire Compagnion:** Investigation, Methodology, Visualization, Writing – review & editing. **Alessandro Matera:** Investigation, Methodology, Visualization, Writing – review & editing. **Daniele Tavernari:** Data curation, Formal analysis, Methodology, Writing – review & editing, Software. **Olivier Campiche:** Methodology, Resources, Validation, Writing – review & editing, Investigation. **Rosa Chiara Paolicelli:** Methodology, Resources, Visualization, Writing – review & editing. **Nadège Zanou:** Formal analysis, Investigation, Methodology, Writing – review & editing. **Bengt Kayser:** Conceptualization, Funding acquisition, Investigation, Methodology, Project administration, Resources, Supervision, Writing – original draft, Writing – review & editing. **Erich Gnaiger:** Funding acquisition, Investigation, Methodology, Project administration, Resources, Supervision, Visualization, Validation, Writing – review & editing. **Nicolas Place:** Data curation, Formal analysis, Funding acquisition, Investigation, Methodology, Project administration, Resources, Supervision, Writing – review & editing.

## Declaration of competing interest

E.G. is the founder and CEO of Oroboros Instruments.

## Data Availability

The published article includes all datasets generated and analyzed in this study. RNA-seq data have been deposited to GEO under accession number GSE225927.

## References

[1] Gnaiger E. (2020). Mitochondrial Pathways and Respiratory Control. An Introduction to OXPHOS Analysis, 5th ed. Bioenerg. Commun..

[2] Glancy B., Balaban R.S. (2021). Energy metabolism design of the striated muscle cell. Physiol. Rev..

[3] Hermansen L., Saltin B. (1969). Oxygen uptake during maximal treadmill and bicycle exercise. J. Appl. Physiol..

[4] Gnaiger E. (2001). Bioenergetics at low oxygen: dependence of respiration and phosphorylation on oxygen and adenosine diphosphate supply. Respir. Physiol..

[5] Donnelly C. (2022). The ABC of hypoxia - what is the norm. Bioenerg. Commun..

[6] Chance B. (1965). Reaction of oxygen with the respiratory chain in cells and tissues. J. Gen. Physiol..

[7] Wagner P.D. (2022). Determinants of maximal oxygen consumption. J. Muscle Res. Cell Motil..

[8] Richardson R.S. (1995). Determinants of maximal exercise VO2 during single leg knee-extensor exercise in humans. Am. J. Physiol..

[9] Calbet J.A. (2015). Limitations to oxygen transport and utilization during sprint exercise in humans: evidence for a functional reserve in muscle O2 diffusing capacity. J. Physiol..

[10] Boushel R. (2011). Muscle mitochondrial capacity exceeds maximal oxygen delivery in humans. Mitochondrion.

[11] Richardson R.S. (2006). Human skeletal muscle intracellular oxygenation: the impact of ambient oxygen availability. J. Physiol..

[12] Chouchani E.T. (2014). Ischaemic accumulation of succinate controls reperfusion injury through mitochondrial ROS. Nature.

[13] Gnaiger E., Mendez G., Hand S.C. (2000). High phosphorylation efficiency and depression of uncoupled respiration in mitochondria under hypoxia. Proc. Natl. Acad. Sci. U. S. A..

[14] Scandurra F.M., Gnaiger E. (2010). Cell respiration under hypoxia: facts and artefacts in mitochondrial oxygen kinetics. Adv. Exp. Med. Biol..

[15] Chandel N.S. (2015). Evolution of mitochondria as signaling organelles. Cell Metabol..

[16] Mottis A., Herzig S., Auwerx J. (2019). Mitocellular communication: shaping health and disease. Science.

[17] Murphy M.P., Chouchani E.T. (2022). Why succinate? Physiological regulation by a mitochondrial coenzyme Q sentinel. Nat. Chem. Biol..

[18] Semenza G.L. (2012). Hypoxia-inducible factors: mediators of cancer progression and targets for cancer therapy. Trends Pharmacol. Sci..

[19] Nunnari J., Suomalainen A. (2012). Mitochondria: in sickness and in health. Cell.

[20] Keeley T.P., Mann G.E. (2019). Defining physiological normoxia for improved translation of cell physiology to animal models and humans. Physiol. Rev..

[21] Simon M.C., Keith B. (2008). The role of oxygen availability in embryonic development and stem cell function. Nat. Rev. Mol. Cell Biol..

[22] Eltzschig H.K., Carmeliet P. (2011). Hypoxia and inflammation. N. Engl. J. Med..

[23] Mihaylova M.M., Shaw R.J. (2011). The AMPK signalling pathway coordinates cell growth, autophagy and metabolism. Nat. Cell Biol..

[24] Harrison D.K. (2015). Cytochrome redox states and respiratory control in mouse and beef heart mitochondria at steady-state levels of hypoxia. J. Appl. Physiol..

[25] Martinez-Reyes I., Chandel N.S. (2020). Mitochondrial TCA cycle metabolites control physiology and disease. Nat. Commun..

[26] Murphy M.P. (2009). How mitochondria produce reactive oxygen species. Biochem. J..

[27] Schmidt O., Pfanner N., Meisinger C. (2010). Mitochondrial protein import: from proteomics to functional mechanisms. Nat. Rev. Mol. Cell Biol..

[28] Giorgi C., Marchi S., Pinton P. (2018). The machineries, regulation and cellular functions of mitochondrial calcium. Nat. Rev. Mol. Cell Biol..

[29] Gnaiger E. (2024). Complex II ambiguities - FADH_2_ in the electron transfer system. J. Biol. Chem..

[30] Komlódi T. (2021). Coupling and pathway control of coenzyme Q redox state and respiration in isolated mitochondria. Bioenerg. Commun..

[31] Kirichok Y., Krapivinsky G., Clapham D.E. (2004). The mitochondrial calcium uniporter is a highly selective ion channel. Nature.

[32] Naszai A. (2019). Ca((2+))N it Be measured? Detection of extramitochondrial calcium movement with high-resolution FluoRespirometry. Sci. Rep..

[33] Glancy B. (2013). Effect of calcium on the oxidative phosphorylation cascade in skeletal muscle mitochondria. Biochemistry.

[34] Vilas-Boas E.A. (2023). Goldilocks calcium concentrations and the regulation of oxidative phosphorylation: too much, too little, or just right. J. Biol. Chem..

[35] Marx S.O., Ondrias K., Marks A.R. (1998). Coupled gating between individual cardiac and skeletal muscle calcium release channels (ryanodine receptors). Circulation.

[36] Marx S.O., Ondrias K., Marks A.R. (1998). Coupled gating between individual skeletal muscle Ca2+ release channels (ryanodine receptors). Science.

[37] Zanou N. (2021). Acute RyR1 Ca(2+) leak enhances NADH-linked mitochondrial respiratory capacity. Nat. Commun..

[38] Jiang B.H. (1996). Hypoxia-inducible factor 1 levels vary exponentially over a physiologically relevant range of O2 tension. Am. J. Physiol..

[39] Garg V. (2021).

[40] Devlin C.M. (2011). Dihydroceramide-based response to hypoxia. J. Biol. Chem..

[41] Richardson R.S. (1999). Evidence of O2 supply-dependent VO2 max in the exercise-trained human quadriceps. J. Appl. Physiol..

[42] Richardson R.S. (1999). Evidence of skeletal muscle metabolic reserve during whole body exercise in patients with chronic obstructive pulmonary disease. Am. J. Respir. Crit. Care Med..

[43] Morales-Alamo D. (2017). Skeletal muscle signaling, metabolism, and performance during sprint exercise in severe acute hypoxia after the ingestion of antioxidants. J. Appl. Physiol..

[44] Morales-Alamo D. (2012). Increased oxidative stress and anaerobic energy release, but blunted Thr172-AMPKalpha phosphorylation, in response to sprint exercise in severe acute hypoxia in humans. J. Appl. Physiol..

[45] Zenebe W.J. (2007). Hypoxia/reoxygenation of isolated rat heart mitochondria causes cytochrome c release and oxidative stress; evidence for involvement of mitochondrial nitric oxide synthase. J. Mol. Cell. Cardiol..

[46] Allen S.P. (1993). Changes in mitochondrial matrix free calcium in perfused rat hearts subjected to hypoxia-reoxygenation. J. Mol. Cell. Cardiol..

[47] Griffiths E.J. (1998). Mitochondrial calcium transporting pathways during hypoxia and reoxygenation in single rat cardiomyocytes. Cardiovasc. Res..

[48] Stern M.D. (1988). Anoxic contractile failure in rat-heart myocytes is caused by failure of intracellular calcium release due to alteration of the action-potential. Proc. Natl. Acad. Sci. U.S.A..

[49] Komlodi T. (2018). Membrane potential and delta pH dependency of reverse electron transport-associated hydrogen peroxide production in brain and heart mitochondria. J. Bioenerg. Biomembr..

[50] Gruszczyk A.V. (2022). Mitochondrial metabolism and bioenergetic function in an anoxic isolated adult mouse cardiomyocyte model of in vivo cardiac ischemia-reperfusion injury. Redox Biol..

[51] Burger N. (2020). A sensitive mass spectrometric assay for mitochondrial CoQ pool redox state in vivo. Free Radic. Biol. Med..

[52] Hernansanz-Agustin P. (2020). Na(+) controls hypoxic signalling by the mitochondrial respiratory chain. Nature.

[53] Mammucari C. (2018). Mitochondrial calcium uptake in organ physiology: from molecular mechanism to animal models. Pflügers Archiv.

[54] Chinopoulos C. (2010). Forward operation of adenine nucleotide translocase during F_0_F_1_-ATPase reversal: critical role of matrix substrate-level phosphorylation. FASEB. J..

[55] Robach P. (2014). Hypoxic training: effect on mitochondrial function and aerobic performance in hypoxia. Med. Sci. Sports Exerc..

[56] Connolly E. (2006). Hypoxia inhibits protein synthesis through a 4E-BP1 and elongation factor 2 kinase pathway controlled by mTOR and uncoupled in breast cancer cells. Mol. Cell Biol..

[57] Howald H. (1990). Muscular exercise at high altitude. Int. J. Sports Med..

[58] Hoppeler H., Klossner S., Vogt M. (2008). Training in hypoxia and its effects on skeletal muscle tissue. Scand. J. Med. Sci. Sports.

[59] Flanigan W.R., Jain I.H. (2022). The Goldilocks Oxygen Principle: not too little and not too much. Nat Cardiovasc Res.

[60] Mela L., Seitz S. (1979). Isolation of mitochondria with emphasis on heart mitochondria from small amounts of tissue. Methods Enzymol..

[61] Komlódi T., Cardoso L.H.D., Gnaiger E. (2021). Isolation of mouse heart mitochondria. Mitochondr. Physiol. Network.

[62] Sumbalová Z., Fontana M., Krumchnabel G. (2016). Isolation of rat brain mitochondria. Mitochondr. Physiol. Network.

[63] Place N. (2015). Ryanodine receptor fragmentation and sarcoplasmic reticulum Ca^2+^ leak after one session of high-intensity interval exercise. Proc. Natl. Acad. Sci. U. S. A..

[64] Gnaiger E. (1995). Control of mitochondrial and cellular respiration by oxygen. J. Bioenerg. Biomembr..

[65] Doerrier C. (2018). High-resolution FluoRespirometry and OXPHOS protocols for human cells, permeabilized fibers from small biopsies of muscle, and isolated mitochondria. Methods Mol. Biol..

[66] Kristiansen G. (2011). Endogenous myoglobin in breast cancer is hypoxia-inducible by alternative transcription and functions to impair mitochondrial activity: a role in tumor suppression?. J. Biol. Chem..

[67] Pesta D., Gnaiger E. (2012). High-resolution respirometry: OXPHOS protocols for human cells and permeabilized fibers from small biopsies of human muscle. Methods Mol. Biol..

[68] Krumschnabel G. (2014). Use of safranin for the assessment of mitochondrial membrane potential by high-resolution respirometry and fluorometry. Methods Enzymol..

[69] Chowdhury S.R. (2015). Simultaneous evaluation of substrate-dependent oxygen consumption rates and mitochondrial membrane potential by TMRM and safranin in cortical mitochondria. Biosci. Rep..

[70] Lowry O.H. (1951). Protein measurement with the Folin phenol reagent. J. Biol. Chem..

[71] Andrews S. (2010).

[72] Dobin A. (2013). STAR: ultrafast universal RNA-seq aligner. Bioinformatics.

[73] Li B., Dewey C.N. (2011). RSEM: accurate transcript quantification from RNA-Seq data with or without a reference genome. BMC Bioinf..

[74] Durinck S. (2009). Mapping identifiers for the integration of genomic datasets with the R/Bioconductor package biomaRt. Nat. Protoc..

[75] Ritchie M.E. (2015). Limma powers differential expression analyses for RNA-sequencing and microarray studies. Nucleic Acids Res..

[76] Robinson M.D., McCarthy D.J., Smyth G.K. (2010). edgeR: a Bioconductor package for differential expression analysis of digital gene expression data. Bioinformatics.

[77] Law C.W. (2016). RNA-seq analysis is easy as 1-2-3 with limma, Glimma and edgeR. F1000Res.

[78] Love M.I., Huber W., Anders S. (2014). Moderated estimation of fold change and dispersion for RNA-seq data with DESeq2. Genome Biol..

[79] Subramanian A. (2005). Gene set enrichment analysis: a knowledge-based approach for interpreting genome-wide expression profiles. Proc. Natl. Acad. Sci. U.S.A..

[80] Liberzon A. (2015). The Molecular Signatures Database (MSigDB) hallmark gene set collection. Cell Syst.

[81] Medina J. (2020). Single-step extraction coupled with targeted HILIC-MS/MS approach for comprehensive analysis of human plasma lipidome and polar metabolome. Metabolites.

[82] Dunn W.B. (2011). Procedures for large-scale metabolic profiling of serum and plasma using gas chromatography and liquid chromatography coupled to mass spectrometry. Nat. Protoc..

[83] Broadhurst D. (2018). Guidelines and considerations for the use of system suitability and quality control samples in mass spectrometry assays applied in untargeted clinical metabolomic studies. Metabolomics.

[84] Tsugawa H. (2014). MRMPROBS suite for metabolomics using large-scale MRM assays. Bioinformatics.

[85] Strojnik V., Komi P.V. (1998). Neuromuscular fatigue after maximal stretch-shortening cycle exercise. J. Appl. Physiol..

[86] Hermens H.J. (2000). Development of recommendations for SEMG sensors and sensor placement procedures. J. Electromyogr. Kinesiol..

[87] Place N. (2010). Muscle fatigue: from observations in humans to underlying mechanisms studied in intact single muscle fibres. Eur. J. Appl. Physiol..

[88] Magistris M.R. (1998). Needle muscle biopsy in the investigation of neuromuscular disorders. Muscle Nerve.

