# Supplementary figures and images for: Functional hypoxia reduces mitochondrial calcium uptake
Source: Redox Biol. 2024 Jan 17;71:103037. doi: 10.1016/j.redox.2024.103037 (PMC10906399; doi:10.1016/j.redox.2024.103037)

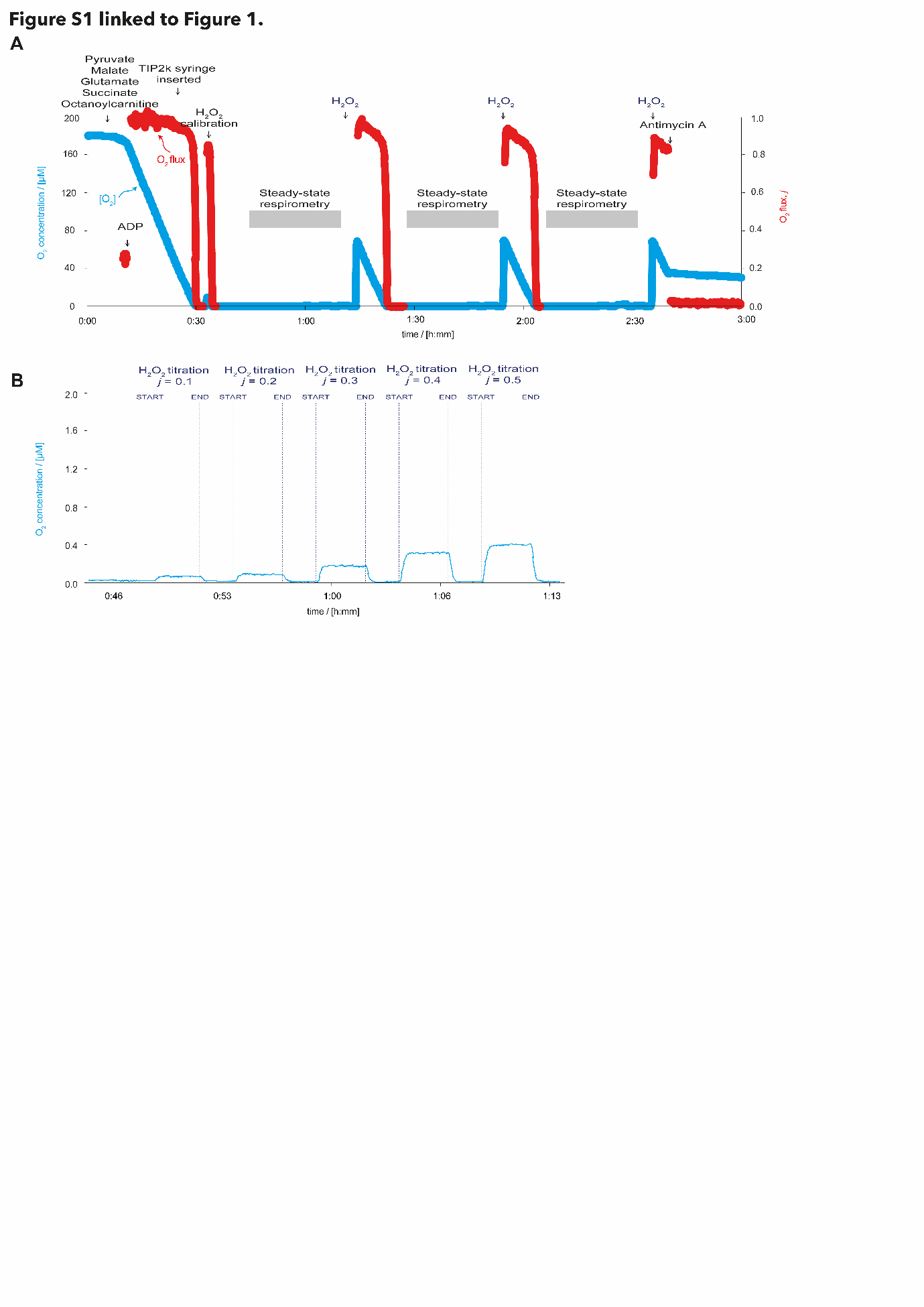


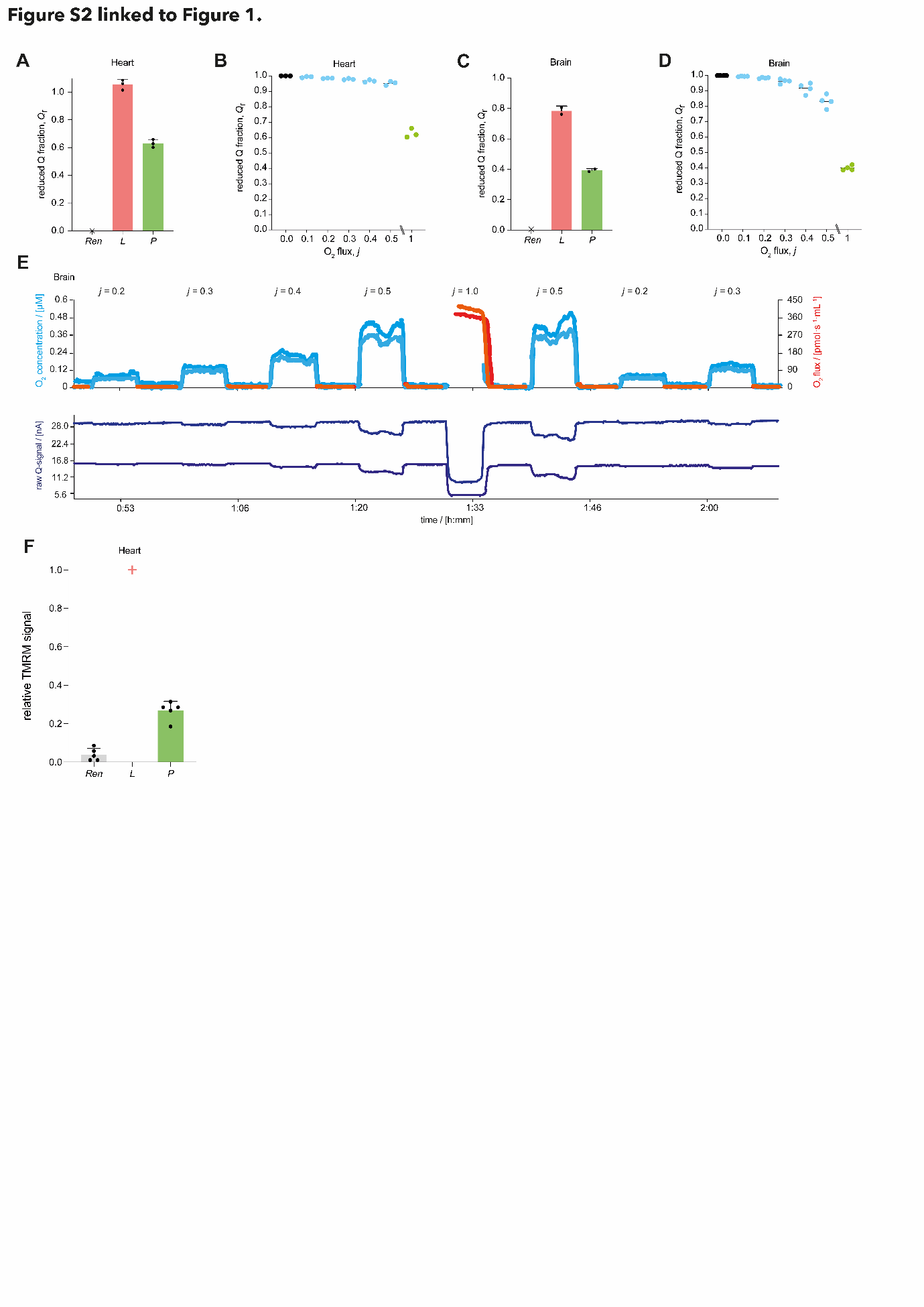


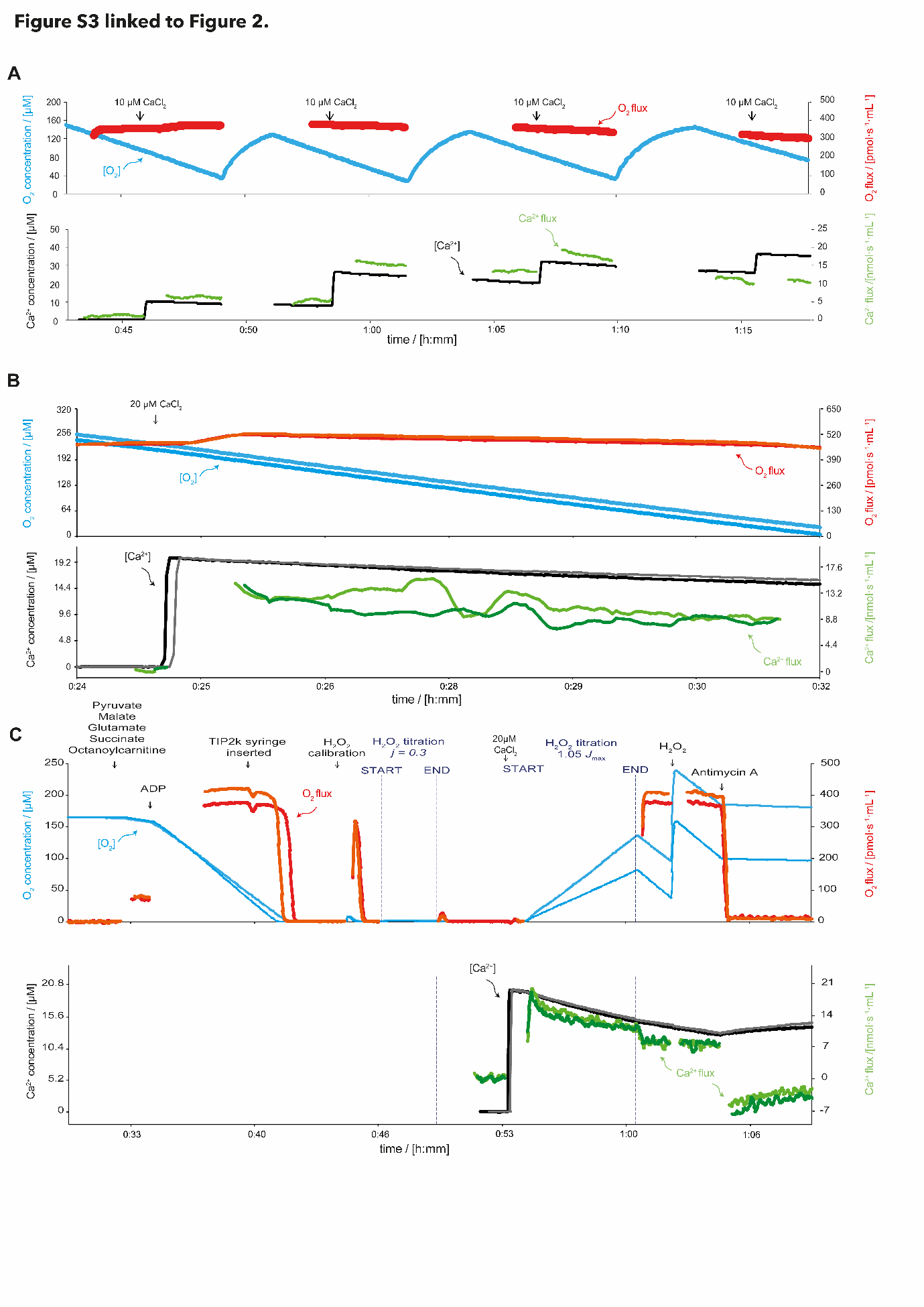


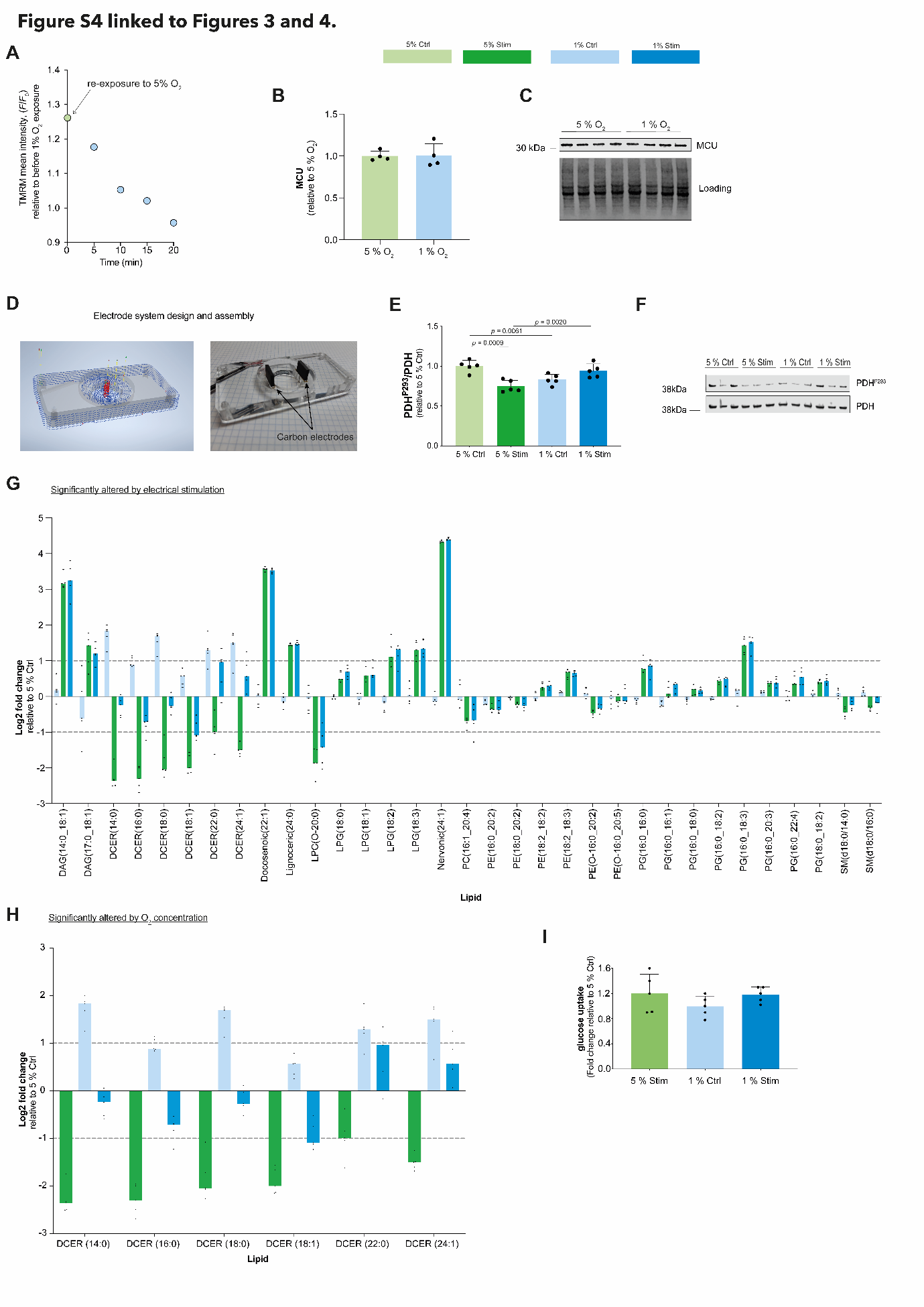


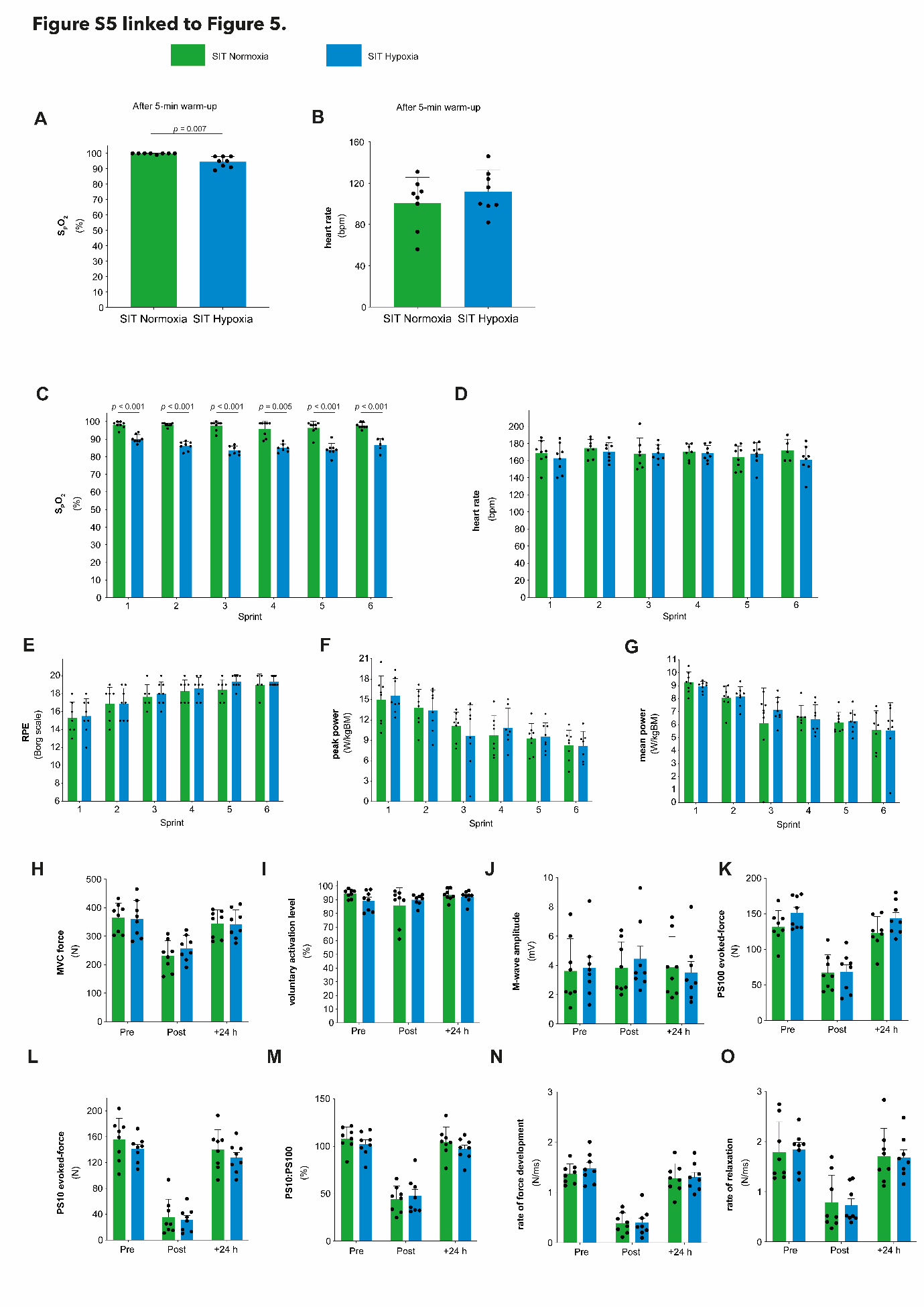

Supplement: Multimedia component 1 [file mmc1.docx]
